# A novel assessment of whole-mount Gleason grading in prostate cancer to identify candidates for radical prostatectomy: a machine learning-based multiomics study

**DOI:** 10.7150/thno.96921

**Published:** 2024-08-01

**Authors:** Jing Ning, Clemens P. Spielvogel, David Haberl, Karolina Trachtova, Stefan Stoiber, Sazan Rasul, Vojtech Bystry, Gabriel Wasinger, Pascal Baltzer, Elisabeth Gurnhofer, Gerald Timelthaler, Michaela Schlederer, Laszlo Papp, Helga Schachner, Thomas Helbich, Markus Hartenbach, Bernhard Grubmüller, Shahrokh F Shariat, Marcus Hacker, Alexander Haug, Lukas Kenner

**Affiliations:** 1 Christian Doppler Laboratory for Applied Metabolomics, Medical University of Vienna, 1090 Vienna, Austria.; 2 Division of Nuclear Medicine, Department of Biomedical Imaging and Image-Guided Therapy, Medical University of Vienna, 1090 Vienna, Austria.; 3 Department of Pathology, Medical University of Vienna, 1090 Vienna, Austria; 4 Central European Institute of Technology, Masaryk University, Brno 62500, Czech Republic.; 5 Department of Biomedical Imaging and Image-guided Therapy, Medical University of Vienna, 1090 Vienna, Austria.; 6 Center for Cancer Research, Medical University of Vienna, 1090 Vienna, Austria.; 7 Center for Medical Physics and Biomedical Engineering, Medical University of Vienna, Vienna, Austria.; 8 Working Group of Diagnostic Imaging in Urology, Austrian Society of Urology, Vienna, Austria.; 9 Department of Urology and Andrology, University Hospital Krems, Karl Landsteiner University of Health Sciences, Krems, Austria.; 10 Department of Urology, Comprehensive Cancer Center, Medical University of Vienna, Vienna, Austria.; 11 Department of Urology, Weill Cornell Medical College, New York, New York.; 12 Department of Urology, University of Texas Southwestern, Dallas, Texas, USA; 13 Hourani Center for Applied Scientific Research, Al-Ahliyya Amman University, Amman, Jordan.; 14 Unit of Laboratory Animal Pathology, University of Veterinary Medicine Vienna, 1210 Vienna, Austria.; 15 Comprehensive Cancer Center, Medical University Vienna, Vienna, Austria.; 16 Center for Biomarker Research in Medicine (CBmed), Graz, Styria, Austria.

**Keywords:** prostate cancer, PSMA, Gleason grading, machine learning, multiomics

## Abstract

**Purpose**: This study aims to assess whole-mount Gleason grading (GG) in prostate cancer (PCa) accurately using a multiomics machine learning (ML) model and to compare its performance with biopsy-proven GG (bxGG) assessment.

**Materials and Methods**: A total of 146 patients with PCa recruited in a pilot study of a prospective clinical trial (NCT02659527) were retrospectively included in the side study, all of whom underwent ^68^Ga-PSMA-11 integrated positron emission tomography (PET) / magnetic resonance (MR) before radical prostatectomy (RP) between May 2014 and April 2020. To establish a multiomics ML model, we quantified PET radiomics features, pathway-level genomics features from whole exome sequencing, and pathomics features derived from immunohistochemical staining of 11 biomarkers. Based on the multiomics dataset, five ML models were established and validated using 100-fold Monte Carlo cross-validation.

**Results**: Among five ML models, the random forest (RF) model performed best in terms of the area under the curve (AUC). Compared to bxGG assessment alone, the RF model was superior in terms of AUC (0.87 vs 0.75), specificity (0.72 vs 0.61), positive predictive value (0.79 vs 0.75), and accuracy (0.78 vs 0.77) and showed slightly decreased sensitivity (0.83 vs 0.89) and negative predictive value (0.80 vs 0.81). Among the feature categories, bxGG was identified as the most important feature, followed by pathomics, clinical, radiomics and genomics features. The three important individual features were bxGG, PSA staining and one intensity-related radiomics feature.

**Conclusion**: The findings demonstrate a superior assessment of the developed multiomics-based ML model in whole-mount GG compared to the current clinical baseline of bxGG. This enables personalized patient management by identifying high-risk PCa patients for RP.

## Introduction

Prostate cancer (PCa) is the second leading cancer-related death in men, with an incidence of nearly 20% worldwide 1. PCa has the highest five-year survival rate (98%) for all stages combined among different tumor types 2. As first-line therapy, radical prostatectomy (RP) has substantially contributed to this phenomenon 3. However, as a consequence of RP, around 31% of patients suffer from urinary incontinence 4, and about 90% suffer from erectile dysfunction 5. Hence, precise identification of individuals who experience minimal clinical advantages but encounter substantial adverse effects in RP is of utmost importance. Currently, the decision on whether to perform RP is mainly determined by biopsy-proven Gleason score (bxGS)^6^. Despite its important role in identifying PCa type, stage, differentiation, and the resulting influence on treatment modality 7, several studies have revealed a strong discrepancy between bxGS and whole-mount GS after RP 8-10. Since whole-mount GS holds a strong association with clinical outcomes 11-13, a more reliable method to assess whole-mount Gleason grading (GG) is needed to accurately identify candidates for RP.

Multiomics provides urologists with comprehensive insights into various aspects of PCa 14, including genetic signatures from genomics, molecular heterogeneity from radiomics, and protein expression from pathomics. Genetic tests of PCa biopsy samples are currently available to predict subsequent disease progression after RP 15. While genomics is nowadays part of the standard repertoire of cancer research approaches, the full prospects of pathomics and positron emission tomography (PET)-based radiomics yet remain to be explored. Radiomics is an emerging field where imaging features are extracted for objective and quantitative tumor characterization 16. Radiomics application on prostate-specific membrane antigen (PSMA) PET scans has shifted clinical PCa research towards a personalized direction 17,18. Recent studies have showcased the capability of pathomics, an approach for the extraction of quantitative features from pathological images, in PCa characterization 19-21. However, no studies have yet leveraged the potential of combining genomics, radiomics, and pathomics. Machine learning (ML) can serve as an ideal platform for the integration of high-dimensional multiomics data.

In this study, we aimed to assess whole-mount GG in PCa accurately using a novel ML approach to identify appropriate candidates for RP and to compare it with biopsy-proven GG (bxGG).

## Materials and Methods

### Study Design

A total of 146 patients with histologically-confirmed PCa from the pilot study of a prospective clinical trial (clinicaltrials.gov NCT02659527) were retrospectively enrolled, all of whom underwent ^68^Ga-PSMA-11 PET/MR scans before RP between May 2014 to April 2020 at the Division of Nuclear Medicine in the Vienna General Hospital. This clinical trial complied with the Helsinki Declaration and its amendments. The inclusion and exclusion criteria were listed in **Supplementary Method M1**. The primary aim of this prospective trial was to improve the detection rate of primary localized PCa using non-invasive PSMA PET/MR in comparison with conventional biopsy. Our study, in contrast to previous work, incorporates radiomics, pathomics and genomics data, offering a more comprehensive analysis while predicting whole-mount Gleason grading rather than the improvement of detection rate. The study was approved by the ethics committee of the Vienna General Hospital (ID: 1649/2016). Each subject gave prior written informed consent.

### Clinical Data Acquisition

Clinical parameters, including age, weight, height, body mass index (BMI), and pre-operative prostate-specific antigen (PSA) levels in serum were collected from the documentation of the clinical trial.

Based on ^68^Ga-PSMA PET/MR images, two nuclear medicine physicians (S.R. and A.H.) with more than 10 years of experience, blinded to the outcome of each patient, assessed six parameters: (1) lesion involvement: whether the tumor affected one or two lobes or was diffusely spread throughout the prostate; (2) lesion position in the anatomy zone: whether the tumor was located in the central zone (CZ), transition zone (TZ), peripheral zone (PZ), anterior fibromuscular stroma (AFS), or it was diffusely distributed (i.e., tumor lesions involving at least two anatomical zones or the whole prostate; (3) extracapsular extension: whether the tumor exceeded the prostate capsule; (4) contact with neurovascular bundles: whether the tumor infiltrated adjacent neurovascular bundles; (5) lymph node (LN) infiltration: whether the tumor infiltrated the pelvic or distant LNs; (6) bone metastasis: whether tumor metastasized to bones.

### Genomics Data Acquisition

Formalin-fixed paraffin-embedded (FFPE) tissue sections (3×10 μm) were obtained from RP samples and DNA extraction was performed. Genomic libraries were prepared and the raw sequencing data were processed. Somatic small variants were identified from paired samples of the tumor and corresponding normal tissue using the SomaticSeq variant caller 22.

Identified variants were annotated using Ensembl's Variant Effect Predictor (VEP) tool. Pathogenicity scores from the evolutionary model of variant effect (EVE) 23, Combined Annotation-Dependent Depletion (CADD) 24, and PolyPhen 25 were annotated and combined into a final pathogenicity metascore for each identified variant after normalization. Pathogenic genetic disruption was computed as the sum of combined pathogenicity scores of all variants in the given gene. Pathway genetic disruption was subsequently computed as the sum of the pathogenicity scores of all genes in each pathway based on the Kyoto Encyclopedia of Genes and Genomes (KEGG).

The tumor mutational burden (TMB) for each sample was computed as the number of identified somatic variants per million base pairs of the sequence region. Copy number variants (CNVs) were called using the CNVkit tool 26 with the set of paired normal samples used as a panel of “normals" in the computation. CNV burden was computed as the ratio of CNV sum size to the sum size of all sequenced regions. More details are described in **Supplementary Method M2 and Supplementary Figure S1**.

### Radiomics Data Acquisition

The imaging protocol was described in a previously published study 27. ^68^Ga-PSMA-11 PET/MR images were acquired and volumes of interest (VOIs) were delineated on PET images with the T2-weighted imaging (T2WI) as anatomical reference. The delineations were performed manually by two nuclear medicine physicians (S.R and A.H) with more than 10 years of diagnostic experience in a slice-by-slice fashion. In instances of differing viewpoints, the physicians reached a consensus through discussion, ensuring precise and accurate identification of the VOIs. PET image intensities were converted to standardized uptake values (SUV) normalized to body weight, and conventional SUV metrics were extracted from VOIs, including SUVmin, SUVmax, SUVmean, SUVpeak, PSMA-tumor volume (PSMA-TV) and total lesion-PSMA (TL-PSMA) 28.

Radiomics features were computed using PyRadiomics 3.0.1 29. All extracted features were compliant with the international biomarker standardization initiative (IBSI) 30. PET images were resampled to an isotropic voxel size of 2x2x2 mm^3^ using B-spline interpolation and bin width was set to 0.3 SUV units. The workflow is shown in **Supplementary Figure S2**.

### Pathomics Data Acquisition

Tissue samples were obtained from FFPE specimens. Tumor areas and normal areas from each sample were delineated on hematoxylin and eosin (H&E)-stained slides by an uro-pathologist with over 30 years of diagnostic experience (L.K.). Three cylindrical cores (diameter: 2.2 mm) were punched from annotated tumor areas and three from normal areas. These cores were transferred to a recipient paraffin block to create an array of tissue samples. The recipient block was sectioned into 2-5 µm thick sections and TMA slides were prepared. The tumor cores were specifically chosen from areas within the RP specimens that presented the most aggressive features upon pathological morphology in order to be most representative of the PCa tissue aggressiveness.

H&E and IHC staining were performed on the TMA slides. PSMA^31^, androgen receptor (AR) 31, Ki-67^32^, PSA^31^, NK3 homeobox 1 (NKX3.1) 31, cyclin-dependent kinase 2 (CDK2) 33,34, cluster of differentiation 3 (CD3) 35, signal transducer and activator of transcription 3 (STAT3) 36, fatty acid synthase (FASN) 31, thyroid hormone receptor beta (TRβ) 37 and interleukin-6 signal transducer (IL6ST)^38^ were selected as targets. The antibodies for IHC staining were listed in **Supplementary Method M3**.

The uro-pathologist (L.K.), blinded to the clinical data, evaluated the GS of each core based on H&E-stained TMA slides. As the punching process effectively mimics the targeted biopsy in clinical routines 39,40, the GS from each core was considered as bxGS to eliminate any time discrepancy. Moreover, the pathologist determined the percentages of strongly, moderately, or weakly stained cells of each core on IHC slides using the modified H-score 41, which was calculated using the formula: ([% of weak staining] × 1) or ([% of moderate staining] × 2) or ([% of strong staining] × 3), yielding a range from 0 to 300 42,43. The average and maximum H-score values from tumor cores were considered representative indicators of different targets' expression levels. The workflow is shown in **Supplementary Figure S3**.

### Reference Standard

As binary ML prediction target, the post-operative International Society of Urological Pathology (ISUP) grading derived from whole-mount samples was split into low-risk (ISUP < 3) and high-risk (ISUP ≥ 3) 44. This aligns with a previous large multicenter study indicating that the best prognostic stratification can be achieved at the threshold of grade three 45. The ISUP grading system allows for better interpretation of morphological patterns and more accurate GG stratification 46,47.

### Machine Learning

The resulting 203 input features included 13 clinical features, 113 radiomics-wide features (107 radiomics features and 6 conventional SUV metrics), 53 genomics features, 23 pathomics features, and 1 feature, namely biopsy-proven ISUP (bxISUP). All the features are listed in **Supplementary Table S1**.

ML was conducted using five classification algorithms, namely k-nearest neighbors (kNN), random forest (RF), extreme gradient boosting (XGB), support vector machine (SVM) and logistic regression (LGR). Robust performance evaluation was performed using 100-fold stratified Monte Carlo cross-validation with 70% of samples in the training set and 30% in the test set. The test set was exclusively used for testing, while a subset of the training data was employed for preprocessing and hyperparameter tuning. Features with more than 30% missing values were excluded. Any remaining missing values were imputed using k-nearest neighbor imputation with distance weighting 48. Features were normalized using z-score. Feature selection was performed using minimum redundancy and maximum relevance (mRMR) 49. Hyperparameter tuning was performed using random search. All procedures, including imputation, normalization, feature selection, and hyperparameter tuning, were performed separately for each fold while fitting on the training set and performing corresponding transformations on the test set to avoid any data leakage. Probability calibration was performed using an isotonic regression.

To ensure maximum transparency of the ML models and to enable the interpretation of decisions made by the applied algorithms, a set of explainable artificial intelligence (XAI) methods were employed, Shapley additive explanations (SHAP), permutation feature importance, and surrogate models. Permutation and SHAP importance both show feature importance, but the calculation of importance values differs 50. Surrogate models are post-hoc explainable artificial intelligence techniques that aim to estimate the predictions of black-box models using a simple and interpretable model. In this study, we extracted this description from the RF model to create a simplified diagnostic workflow (decision tree). Further details on ML are described in **Supplementary Method M4**.

### Statistical Analysis

The Python 3 package-scipy package 1.11.4 was used for statistical analysis. Quantitative metrics were calculated as mean ± standard deviation (SD), and categorical variables as percentages. Mann-Whitney test was used for comparison between quantitative variables. The chi-square test was used for comparison between categorical variables. All *p* values were tested as two-tailed. *P* values less than 0.05 were regarded as statistically significant.

## Results

### Data Overview

**Figure 1:**
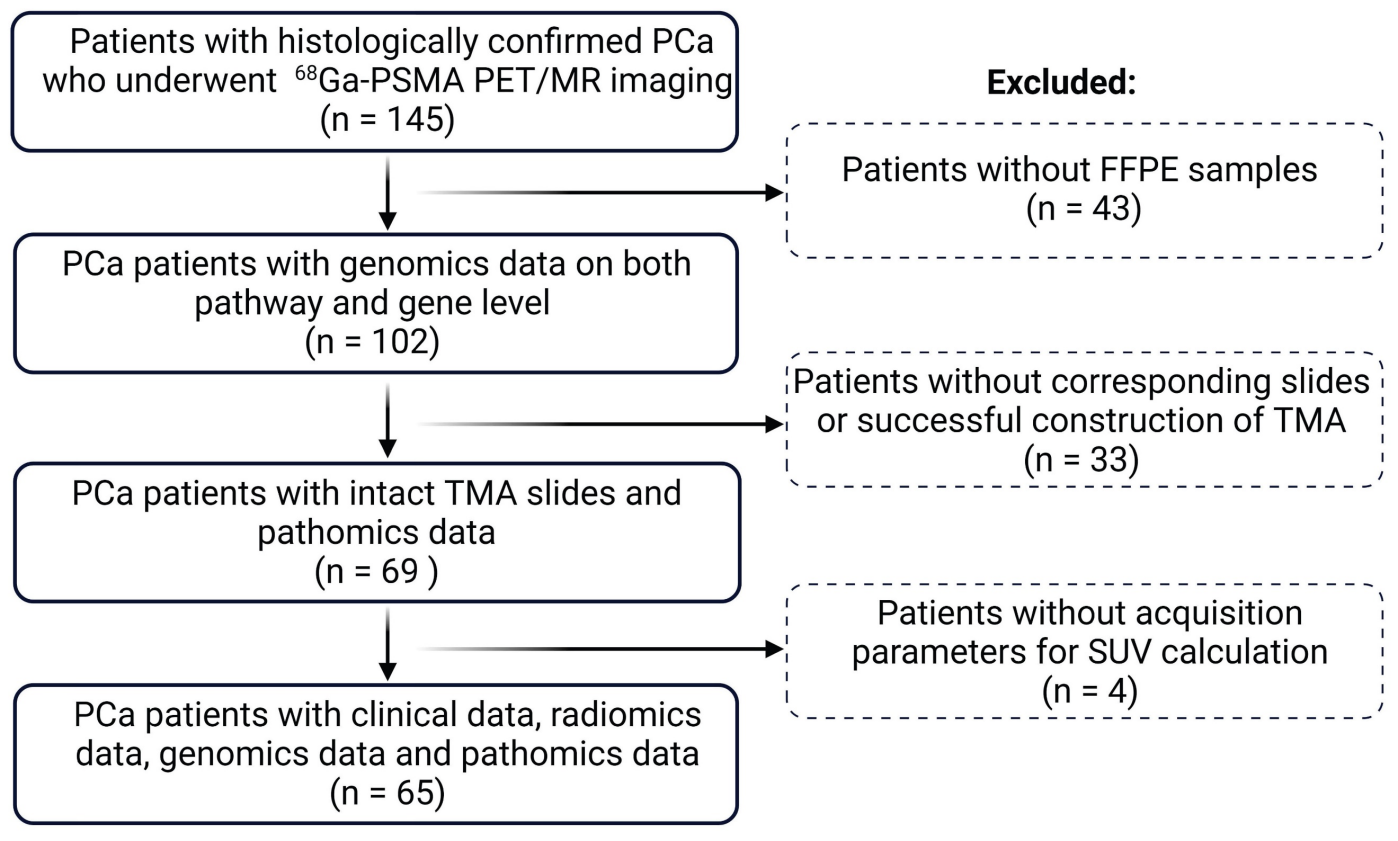
** Flowchart of the study cohort.** PCa: prostate cancer; TMA: tumor microarray; FFPE: formalin-fixed paraffin-embedded; SUV: standardized uptake value.

A total of 65 PCa patients (age: 64 ± 7.6) with clinical, genomics, radiomics, and pathomics data were included for final analysis following the cohort flow chart in **Figure 1**. Of these patients, 28 (43%) patients (age: 62.4 ± 7.9) were categorized as low risk (ISUP < 3) and 37 (57%) patients (age: 65.2 ± 7.1) as high risk (ISUP ≥ 3). Clinical and imaging parameters of both groups are shown in **Table 1**.

**Figure 2:**
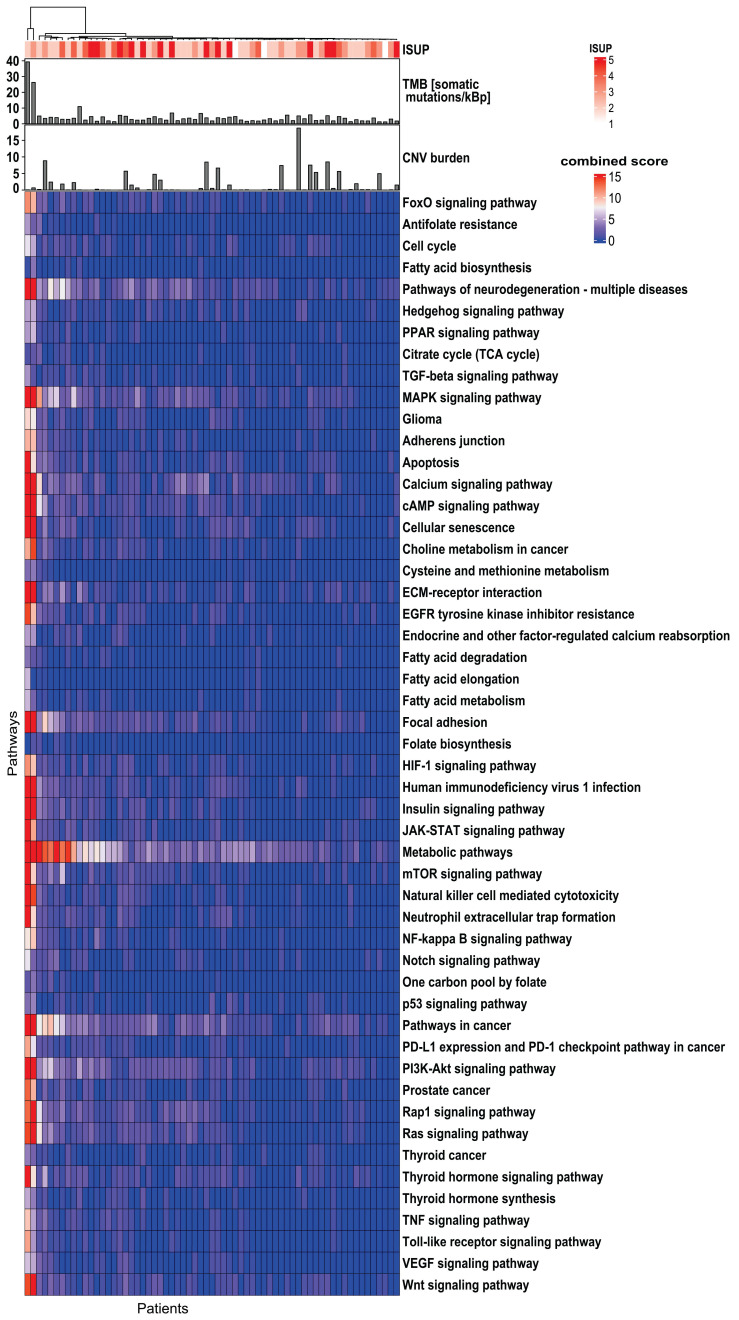
**Genomics profile indicates the heterogeneity of the 51 investigated biological pathways in 65 PCa patients.** The top bar shows TMB and CNV burden distribution. The top panel shows the correlation of genes/pathways mutation profile with ISUP groups. The top dendrogram shows the clustering patterns of genes/pathways based on their mutation profiles. TMB: tumor mutational burden; CNV: copy number variant; ISUP: International Society of Urological Pathology; PCa: prostate cancer.

Based on the KEGG database, a total of 10,305 genes were identified in the exome of DNA. The overall mutation frequency was low, with only 29 genes mutated in ≥10% of patients (**Supplementary Figure S4**). No significant correlation (*p* ≥0.05) was found between any of the gene mutations and TMB, CNV burden, or whole-mount ISUP. Due to the sparse distribution of gene mutations, only pathway-level genomics features were employed for the subsequent ML analysis (Figure 2). Among 341 pathways annotated in the KEGG database, 51 pathways were selected due to their association with PCa tumorigenesis, aggressiveness, progression, or metastasis. The literature is listed in **Supplementary Table S2** to show the predictive potential of 51 pathways.

In total, 107 radiomics features were extracted and categorized into shape (n=14), histogram (n=18), and texture (n=75) features. Texture features included 24 gray level co-occurrence matrix (GLCM), 16 gray level run length matrix (GLRLM), 16 gray level size zone matrix (GLSZM), 14 gray level dependence matrix (GLDM) and 5 neighboring gray-tone difference matrix (NGTDM) features. Features are categorized in **Supplementary Table S3**.

**Figure 3:**
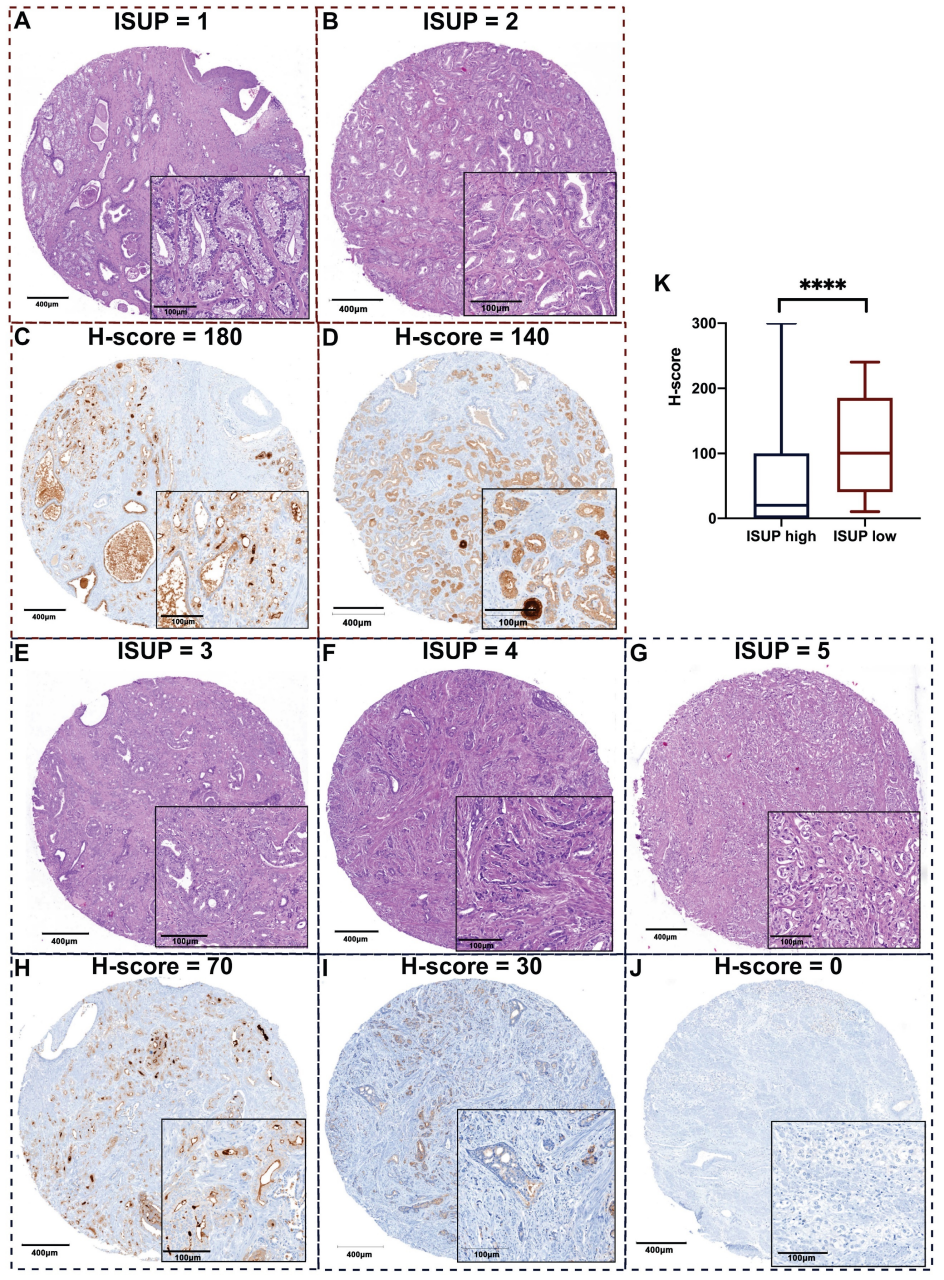
** Representative images of H&E staining and PSA staining on TMA slides revealing less PSA expression when PCa tissue is more aggressive.** A. Representative images of H&E staining for each ISUP grade: a. Patient 1, GS 6 (3+3); b. Patient 2, GS 7 (3+4); c. Patient 3, GS 7 (4+3); d. Patient 4, GS 8 (4+4); e. Patient 5, GS 9 (4+5); according to ISUP consensus 2019. B. Representative images of PSA expression in each ISUP grade core. a. Patient 1, high PSA expression; b. Patient 2, relatively high PSA expression; c. Patient 3, moderate PSA expression; d. Patient 4, relatively low PSA expression; d. Patient 5, negative PSA expression. The corresponding H&E core and PSA core are from the same cylinder of the same patient. The scale bars of the overview core and enlarged details are 400 μm and 100 μm respectively. C. The maximum H-score of PSA is significantly different between ISUP high and low groups (*p* < 0.0001). TMA: tumor microarray; PSA: prostate-specific antigen; ISUP: International Society of Urological Pathology; GS: Gleason score.

ISUP grading was determined by H&E-staining with morphological details depicted in **Figure 3A**. Representative images of PSA expression between ISUP high and low groups are shown in **Figure 3B**. After statistical analysis of the whole cohort, we found that the maximum H-score of PSA was the most distinguished biomarker, and its value in high-risk PCa was lower than that of the low-risk group.

### Machine Learning Performance

**Figure 4:**
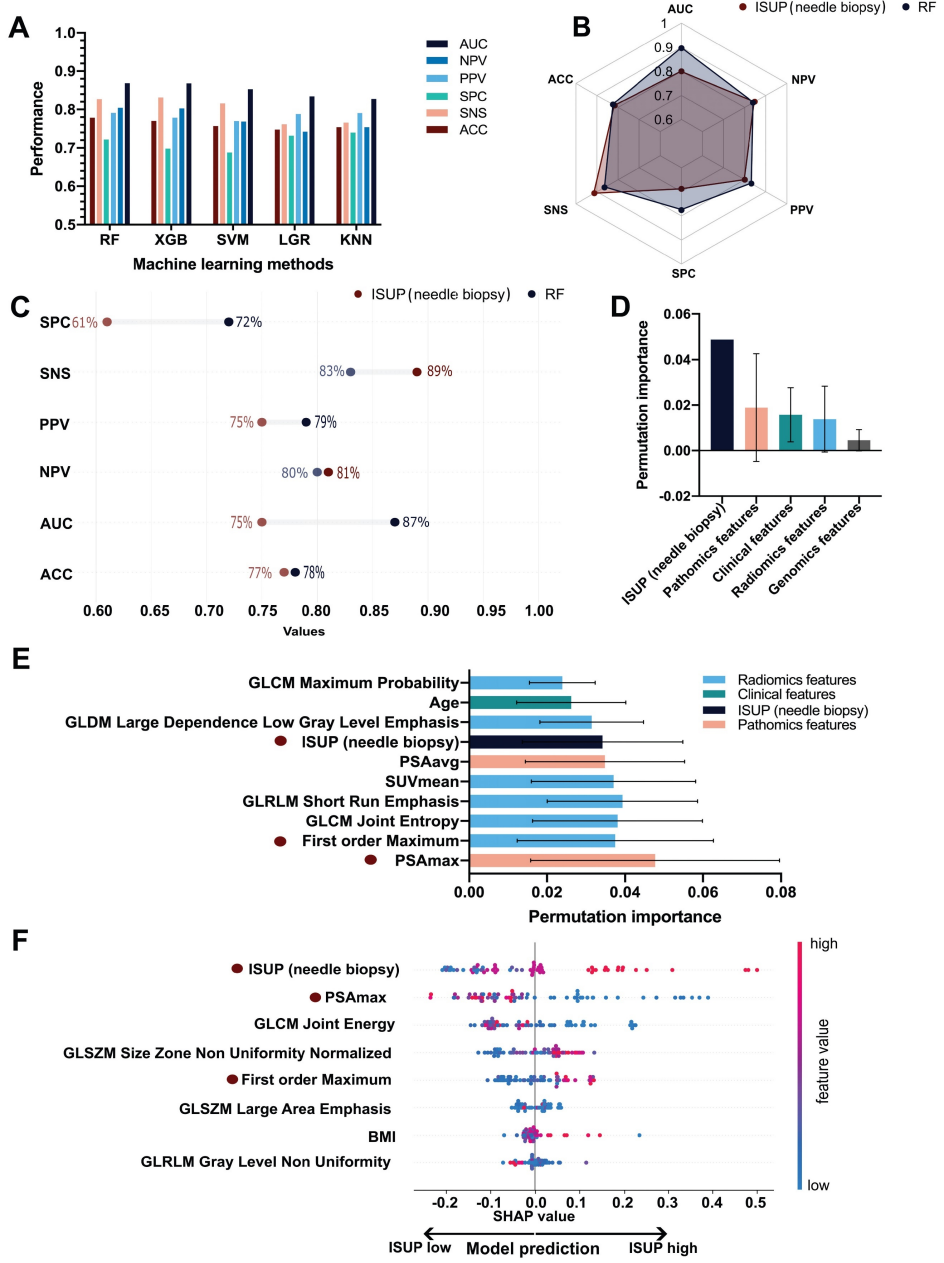
** ML Performance of the ISUP prediction in PCa.** A. Performance comparison of the five ML algorithms (KNN, RF, SVM, LGR, XGB). Ranked by AUC, the RF model had the best performance. B. Overall comparison of different performance metrics between the RF model and ISUP derived from needle biopsy. C. Comparison of the mean permutation importance between different types of features. D. Detailed comparison of different performance metrics between the RF model and ISUP derived from needle biopsy. E. The top 10 performing features in ISUP prediction based on permutation importance over all cross-validation folds. F. SHAP importance of the eight features included in the final RF model trained on the entire dataset. Each dot represents a single patient and higher feature values are labeled as red while lower values are blue. The increasing positive SHAP values are indicative of the model's tendency to predict high ISUP while decreasing SHAP values indicate the tendency of the model to predict low ISUP. KNN: k-nearest neighbors; RF: random forest; XGB: extreme gradient boosting; SVM: support vector machine; LGR: logistic regression; AUC: area under the curve; ACC: accuracy; SNS: sensitivity, SPC: specificity; PPV: positive predictive value; NPV: negative predictive value; ML: machine learning; SUVmean: mean standardized uptake value; PSAmax: maximum H-score of PSA expression on three cores of TMA slides; PSAavg: average H-score of PSA expression on three cores of TMA slides.

The five ML models (KNN, RF, SVM, LGR, XGB) were compared based on area under the curve (AUC), accuracy (ACC), sensitivity (SNS), specificity (SPC), positive predictive value (PPV) and negative predictive value (NPV), as depicted in **Figure 4A**. In terms of AUC, the RF model achieved the highest performance (**Supplementary Table S4**). The AUC, ACC, SNS, SPC, PPV, and NPV of the RF model were 0.87 (95%confidence interval ([CI], 0.85-0.89), 0.78 (95%CI, 0.76-0.80), 0.83 (95%CI, 0.80-0.86), 0.72 (95%CI, 0.68-0.76), 0.79 (95%CI, 0.77-0.81) and 0.80 (95%CI, 0.77-0.83) respectively.

The performance metrics of needle biopsy AUC, ACC, SNS, SPC, PPV, and NPV were 0.75, 0.77, 0.83, 0.61, 0.75, and 0.80 respectively. In comparison, the performance of RF showed an increase in AUC, ACC, SNS, and NPV by 12%, 1%, 11%, and 1%, respectively while SNS and PPV decreased by 6% and 4% (**Figure 4B. 4C**).

A total of 73 features were selected in the validation procedure, consisting of 1 feature, namely bxISUP, 5 clinical features, 12 gene-level genomics features, 43 radiomics-wide features and 13 pathomics features. After comparison of the mean permutation importance of different types of features, bxISUP was identified as the most attributable feature, followed by pathomics, clinical, radiomics, and genomics features (**Figure 4D**). Ranked by permutation importance value, the ten most important features included six radiomics features, three pathomics features, one clinical feature, and biopsy-derived ISUP (**Figure 4E**). More details were described in **Supplementary Results R1-3**.

SHAP importance revealed bxISUP as the most predictive feature, followed by maximum H-score of PSA, and texture/histogram-based radiomics features. **Figure 4F** shows the top 8 features and their SHAP importance values between high and low ISUP groups. In the ISUP high group, ISUP derived from needle biopsy tends to be higher, PSA is less expressed on IHC slides and GLCM Joint Energy values are lower compared to the ISUP low group.

**Figure 5:**
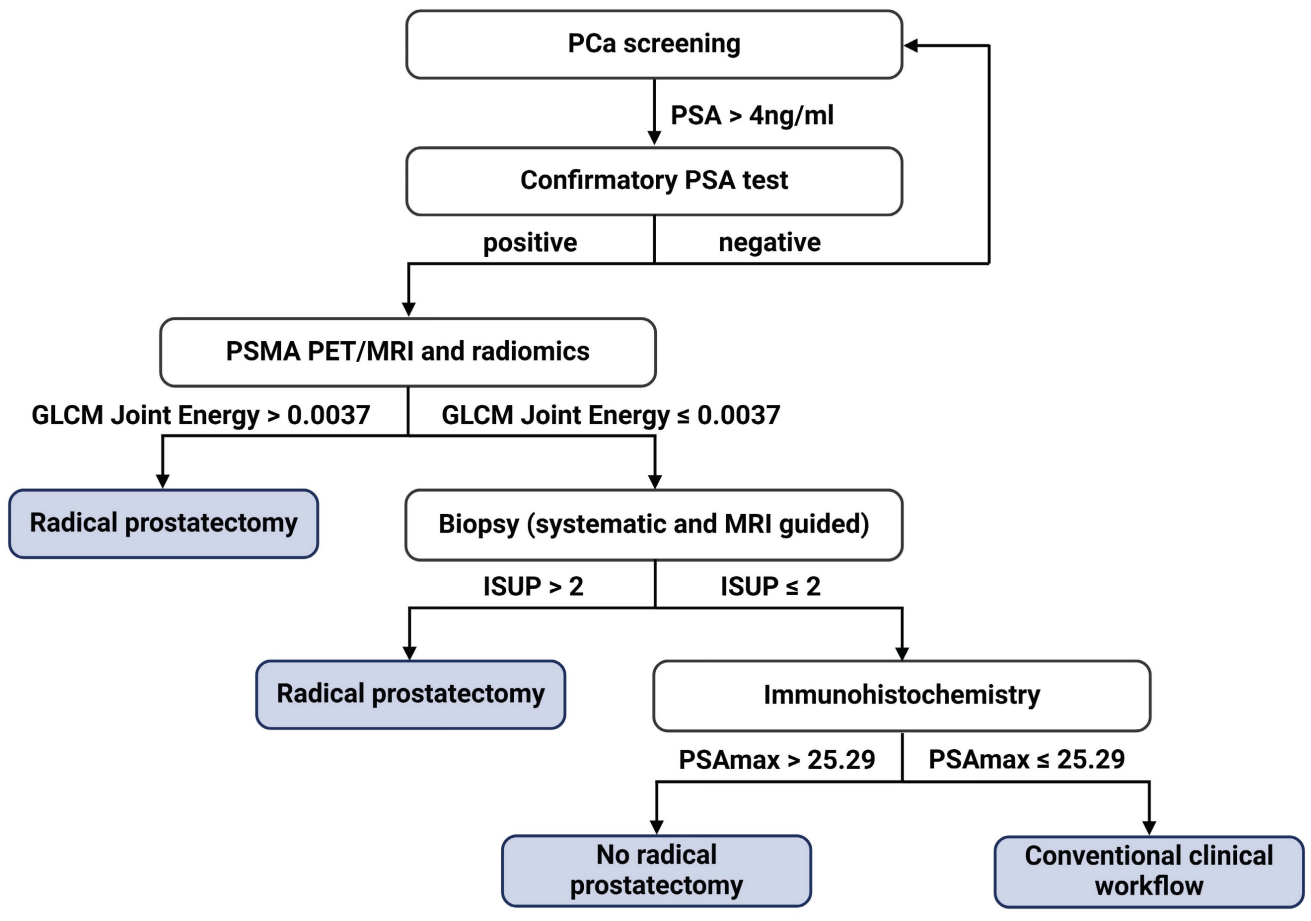
** Proposed diagnostic flowchart for prostate cancer (PCa) management.** PSAmax: maximum H-score of PSA expression on three cores of TMA slides; PSA: prostate-specific antigen.

A surrogate model was established to provide a simplified diagnostic workflow describing the more complex ML model (RF). The resulting simplified diagnostic workflow included three features, which were GLCM_Joint Energy, PSAmax_IHC, and bxISUP, which achieved a performance of AUC 0.89 in estimating the output of the complex ML model (**Supplementary Figure S5**). Based on the surrogate model, the ML-based workflow is incorporated in the clinical diagnostic scheme (**Figure 5**). We further performed three analyses, each using a combination of two feature types, including genomics, pathomics, and radiomics as input. AUC values ranged between 0.84 and 0.89 with the full performance metrics for the individual analyses shown in **Table 2**.

## Discussion

In this study, we integrated clinical, imaging, pathomics, and genomics data for the ML-based GG prediction in PCa and demonstrated the superiority of the ML approach over the clinical standard of bxGG assessment. Furthermore, we developed a simplistic and interpretable diagnostic workflow, enabling a software-independent step-by-step procedure for the identification of high-risk patients instead of running the ML software. This makes validation and integration of the presented findings substantially easier since the repeatability and adaptability of ML models are major hurdles for the translation of ML-based software into clinical settings 51,52.

Numerous published multiomics studies in PCa aimed to guide clinical decision-making by directly inferring clinically relevant outcomes and parameters 53-56. However, most of them focused on predictors from genomics, epigenomics, transcriptomics, and proteomics, omitting image-based predictors, which is problematic given that imaging features have been shown to be important for GS prediction 57-59. This study addresses this gap by integrating not only PSMA PET radiomics with genomics features but also additional pathomics and clinical features. Thus, by leveraging diverse data sources, the ML model capitalized on comprehensive and complementary underlying information, facilitating more accurate GG assessment.

Despite the slight decrease in SNS and NPV, the AUC, ACC, SPC, and PPV of the ML model were superior to those of needle biopsy. The increased specificity, in comparison with the current clinical standard, indicates that the ML model has the ability to identify low ISUP patients accurately, which is aligned with our goal to avoid unnecessary interventions. Also, the high PPV is indicative of our study's reliability in identifying high-risk patients to provide timely and appropriate treatment.

Despite a discrepancy between bxGG and whole-mount GG, bxISUP was among the most important features in our analysis. Especially when combining needle biopsy with additional features such as PSA, ML outperformed the current clinical standard of bxGG substantially. Additionally, our findings unveil that PSAmax as the most important feature in predicting whole-mount GG based on two importance measurement algorithms. PSAmax represents the maximum H-score of the tumor tissue in needle biopsy and our results denote the more aggressive PCa is, the less PSA the tumor tissue expresses. This is consistent with the study that also explored the correlation between PSA H-scores and GG using TMA slides 60. In line with other investigations 61, our study identified the first-order radiomics feature Maximum as an important feature in GG prediction. This is because this histogram-based feature, similar to the conventional SUVmax, manifests the highest uptake of ^68^Ga-PSMA-11.

Of note, our ML model provides a simplified surrogate diagnostic workflow by combining the radiomics feature GLCM_Joint Energy, PSAmax_IHC, and ISUP in needle biopsy. Following the corresponding decision tree, urologists can select appropriate candidates for RP, which has the potential to revolutionize the diagnostic workflow of PCa. In addition to the two previously mentioned common features, the decision tree also includes GLCM_Joint Energy, a radiomics feature indicative of homogeneous patterns within PCa lesions. The lower Energy value means more heterogeneity within the tumor. Our results demonstrate that PCa with higher GG is more heterogeneous, as previously suggested by a study that identified the transcriptomic heterogeneity of GG 5 groups in a large dataset 62.

Despite the promising results, our study still has several limitations. First, due to its design as a retrospective multiomics study, not all the required parameters were available in some patients, resulting in a relatively small number of subjects for analysis. Second, due to the complexity and unique nature of our study, incorporating an independent validation cohort from another center poses significant challenges, particularly in the retrospective collection of high-dimensional datasets that are consistent with the ones used in our study. However, to ensure the robustness and validity of our findings, employed a rigorous 100-fold Monte Carlo cross-validation scheme, which enhanced the robustness, generalizability, and reduced bias of our study. Third, ML models in medical imaging, specifically in nuclear medicine, are known to suffer from center-specific variabilities, reducing the reproducibility of radiomics features 63-65. Consequently, external validation is needed to verify the reliability of the developed approach in the future.

In conclusion, the presented multiomics ML model poses a promising advance in GG assessment for the improved stratification of PCa patients for RP. Our findings have the potential to substantially impact clinical decision-making and personalized management of PCa patients.

## Supplementary Material

Supplementary materials, figures and tables.

## Figures and Tables

**Table 1:** ** Comparison of clinical and imaging parameters between the ISUP low (ISUP<3) group and ISUP high (ISUP≥3) group.** Continuous data are expressed as mean ± standard deviation (SD); categorical variables are presented as numbers and percentages.

Parameters		Low risk (ISUP < 3)	High risk (ISUP ≥ 3)	*p* value
**Clinical Parameters**				
Age (years)		62.4 (7.9)	65.2 (7.1)	0.14
Weight (kg)		80.5 (10.9)	86.7 (11.1)	0.01
Height (m)		1.8 (0.1)	1.8 (0.1)	0.25
BMI (kg/m^2^)		25.8 (2.8)	27.4 (3.3)	0.05
PSA-pre OP (µg/l)		9.4 (8.0)	55.4 (135.0)	<0.001
Pre-OP therapy	*No*	27 (96.43%)	30 (81.08%)	0.21
	*Yes*	1 (3.57%)	5 (13.51%)	
	*NA*	0 (0%)	2 (5.41%)	
**Image-based Parameters**				
Lesion involvement	*One lobe*	13 (46.43%)	20 (54.05%)	0.23
	*Two lobes*	5 (17.86%)	3 (8.11%)	
	*Whole prostate*	0 (0%)	2 (5.41%)	
	*NA*	10 (35.71%)	12 (32.43%)	
Lesion position in anatomy zone*	*CZ*	1 (3.57%)	0 (0%)	0.49
	*TZ*	2 (7.14%)	1 (2.7%)	
	*PZ*	12 (42.86%)	18 (48.65%)	
	*AFS*	0 (0%)	1 (2.7%)	
	*Diffusion*	2 (7.14%)	5 (13.51%)	
	*NA*	11 (39.29%)	12 (32.43%)	
Extracapsular extension	*No*	17 (60.71%)	11 (29.73%)	<0.001
	*Yes*	1 (3.57%)	14 (37.84%)	
	*NA*	10 (35.71%)	12 (32.43%)	
Contact to neurovascular bundles	*No*	18 (64.29%)	20 (54.05%)	0.06
	*Yes*	0 (0%)	5 (13.51%)	
	*NA*	10 (35.71%)	12 (32.43%)	
Lymph node infiltration	*No*	17 (60.71%)	17 (45.95%)	0.03
	*Yes*	1 (3.57%)	9 (24.32%)	
	*NA*	10 (35.71%)	11 (29.73%)	
Bone metastasis	*No*	17 (60.71%)	21 (56.76%)	0.38
	*Yes*	1 (3.57%)	4 (10.81%)	
	*NA*	10 (35.71%)	12 (32.43%)	
Clinical T staging	*cT2a*	3 (10.71%)	4 (10.81%)	0.03
	*cT2b*	5 (17.86%)	2 (5.41%)	
	*cT2c*	8 (28.57%)	4 (10.81%)	
	*cT3a*	1 (3.57%)	3 (8.11%)	
	*cT3b*	1 (3.57%)	12 (32.43%)	
	*cT3a+b*	0 (0%)	1 (2.7%)	
	*cT4*	0 (0%)	1 (2.7%)	
	*NA*	10 (35.71%)	10 (27.03%)	

Value in the bracket is standard deviation for numeric data and percentage for categorical data *CZ: central zone; TZ: transition zone; PZ: peripheral zone; AFS: anterior fibromuscular stroma; Diffusion means PCa lesions involve any two/three anatomy zones or the whole prostate; NA: not applicable.

**Table 2:** Performance for different input feature type combinations.

Feature types	ACC	SNS	SPC	PPV	NPV	BACC	AUC
Genomics and pathomics	0.805	0.830	0.775	0.822	0.820	0.803	0.893
Radiomics and genomics	0.727	0.743	0.708	0.770	0.716	0.726	0.835
Radiomics and pathomics	0.781	0.820	0.735	0.795	0.803	0.778	0.874
Radiomics, genomics and pathomics	0.779	0.827	0.722	0.791	0.804	0.774	0.869

ACC: accuracy; SNS: sensitivity; SPC: specificity; PPV: Positive predictive value; NPV: Negative predictive value; BACC: Balanced accuracy; AUC: Area under the receiver operating characteristic curve.
