# Supplementary material for: A novel assessment of whole-mount Gleason grading in prostate cancer to identify candidates for radical prostatectomy: a machine learning-based multiomics study
Source: Theranostics. 2024 Aug 1;14(12):4570–81. doi: 10.7150/thno.96921 (PMC11373617; doi:10.7150/thno.96921)
Supplement: Supplementary file 1 — Supplementary materials, figures and tables. [file thnov14p4570s1.pdf]

Supplementary Material—Methods

M1: Inclusion and exclusion criteria of these two clinical trials:

(1) Inclusion criteria

Clinical suspicion of prostate cancer:

- blood PSA level > 4.0 ng/ml and/or
- free-to-total PSA ratio <22% and/or
- progressive rise of PSA levels in two consecutive blood samples despite antibiotics

(2) Exclusion criteria

- antiandrogen therapy
- prostate needle biopsy <21 days before PET/MRI
- known active secondary cancer
- endorectal coil not applicable (e.g. anus praetor with short rectal stump)
- known active prostatitis (e.g. painful DRE)
- known anaphylaxis against gadolinium-DOTA
- patient’s written informed consent not given
- Needle biopsy and/or prostatectomy compound not available for histology immunohistochemistry

M2: Genomics Data Acquisition

FFPE tissue processing and DNA isolation Formalin-Fixed Paraffin-Embedded (FFPE) tissue sections (3×10 μm) derived from the RP were prepared from the archival blocks. The sections were deparaffinized using xylene and rehydrated through a series of ethanol washes. DNA extraction from FFPE tissues was performed using the EZ1 DNA tissue kit following the manufacturer's instructions. The extracted DNA was quantified using a spectrophotometer and assessed for quality using agarose gel electrophoresis.

Library preparation Library preparation was carried out using the xGen™ DNA Library Prep EZ UNI (IDT) with xGen™ CS adapters (IDT) containing UMIs. FFPE DNA was first repaired using NEBNext® FFPE DNA Repair Mix (New England Biolabs) according to the original protocol. A total of 300 ng DNA for tumor samples and 100 ng DNA for normal samples was used as input. After index PCR and library purification, the KAPA HyperCapture Reagent Kit (Roche) was used to enrich exome sequences with KAPA HyperExome Probes (Roche) and backbone sequences for CNVs identification with KAPA HyperCap Custom Probes (Roche). The library quantity and size distribution were verified using the QuantiFluor dsDNA System (Promega) and High Sensitivity NGS Fragment Analysis Kit (Agilent Technologies). The finalized library pool was sequenced on NovaSeq 6000 (Illumina) using SP Reagent Kit v1.5 200 cycles (Illumina) in paired-end mode. Raw sequence data in FASTQ format were generated and stored for subsequent analysis.

Data Processing and Variant Calling The raw sequencing data were pre-processed to remove adapter sequences and trim low-quality bases. The cleaned reads were aligned to the human reference genome GRCh38 using BWA alignment software [1]. Duplicate reads were identified and removed using the UMI-aware version of MarkDuplicates from Picard Tools [2]. Somatic small variants, including single nucleotide variants (SNVs) and small insertions and deletions (indels), were identified from paired tumor and corresponding normal tissue samples using the SomaticSeq variant caller [3], a meta-caller that aggregates calls from multiple tools, including Strelka2 [4], VarDict [5], MuTect [6], SomaticSniper [7], LoFreq [8], MuSE [9], and VarScan2 [10].

Variant Annotation and Filtering Identified variants were annotated using Ensembl's Variant Effect Predictor (VEP) tool [11], utilizing its full annotation cache. Pathogenicity scores from the Evolutionary Model of Variant Effect (EVE) [12], Combined Annotation-Dependent Depletion (CADD) [13], and PolyPhen-2 [14] were annotated, alongside cancer-specific annotations from clinical databases such as fOne, MD Anderson, TruSight Oncology, and the Cancer Gene Census (CGC). Specific filtering criteria were applied to identify probable true positive somatic variants and mitigate potential FFPE DNA artifacts:

- Minimal tumor variant depth of 35.
- Variants with a tumor variant allelic frequency (tVAF) lower than 2% were removed.
- Variants with tVAF between 2% and 5% were retained only if the variant read depth was more than 100 and there was at least one record for the variant in the COSMIC database.
- Variants not meeting these criteria were removed as potential false positives.
- Variants identified in the GnomAD or 1000 Genomes database with a population MAF of non-Finnish Europeans higher than 2%, and not having a record in the COSMIC database, were filtered out as potential germline variants.

Genetic Disruption Calculation The pathogenicity scores of CADD were normalized to a zero to one range by mapping the raw values to a logistic distribution. Scores were prioritized from best to worst (EVE, CADD, PolyPhen-2) according to the most recent benchmarks in the EVE paper. The highest available score was then selected as the combined pathogenicity score for every variant. Pathogenic genetic disruption of each gene was computed as the sum of combined pathogenicity scores of all variants within that gene. Pathway genetic disruption was subsequently computed as the sum of the genetic disruption of all genes in each pathway, derived from the Kyoto Encyclopedia of Genes and Genomes (KEGG) [15].

Genomic Features Tumor mutational burden (TMB) for each sample was computed as the

number of identified coding non-synonymous single nucleotide variants per million base pairs of the sequenced region. Copy number variants (CNVs) were called using CNVkit [16] with a set of paired normal samples used as a panel of normals for the computation. Any region with a predicted copy number differing from 2 was considered a CNV. The sum size of all CNVs was computed for each sample, and CNV burden was calculated as the ratio of the CNV sum size to the sum size of all sequenced regions.

The schematic workflow of genomics data acquisition is shown in **Supplementary Figure S1**.

M3: Antibodies used for IHC staining

The following antibodies were respectively used: PSMA (clone EP192, Cell Marque 327R-18, rabbit monoclonal, ready to use), AR (clone EPR1535(2), Abcam ab133273, rabbit monoclonal, 1:100 dilution), Ki-67 (clone rabbit anti-human, Novocastra, NCL-KI67-p, Rabbit Polyclonal,dilution 1:1000), PSA (clone ER-PR8, DAKO M750, mouse monoclonal, 1:20 dilution), NKX3.1 (clone N/A, Biocare Medical CP4228, Rabbit Polyclonal, 1:100 dilution), CDK2 (clone E8J9T, Cell signaling, #18048, rabbit monoclonal, dilution 1:250), CD3 (clone SP7, Neomarkers RM9107, rabbit monoclonal, dilution 1:150), STAT3 (clone 124H6, Cell signaling, #9139, mouse monoclonal, dilution 1:100), FASN (clone C20G5, Cell signaling, #3180, rabbit monoclonal, dilution 1:80), TRβ (clone 2386, Rockland 209-301-A96, mouse monoclonal, dilution 1:100), gp130 (clone E-8, Santa Cruz Biotechnology sc-376280, mouse monoclonal, dilution 1:25).

Details on how the whole-mount pathology performed:
After surgical removal, the prostate was fixed in formalin and sectioned at approximately 3 to 4 mm intervals on whole-mount slides. These distances were chosen to ensure comprehensive coverage and detailed examination of the gland, which is standard practice in prostate pathology. Each slide was stained with haematoxylin and eosin (H&E) for detailed tissue analysis. A specialist urological pathologist (L.K.) examined these slides under the microscope, focusing on the grading of the cancer, tumour margins and possible extraprostatic extension. This systematic approach enabled an accurate assessment of tumor characteristics, which are essential for effective staging and treatment decisions.
The schematic workflow of pathomics data acquisition is shown in **Supplementary Figure S3**.

M4: Technical details on machine learning.

ML was conducted using five classification algorithms, namely k-nearest neighbours (kNN), random forest (RF), extreme gradient boosting (XGB), support vector machine (SVM) and logistic regression (LGR). Robust performance evaluation was performed using 100-fold stratified Monte Carlo cross-validation with 70% of samples in the training set and 30% in the test set. The test set was exclusively used for testing while a subset of the training data was employed for preprocessing and hyperparameter tuning. Feature standardization was performed using z-scaling. Features were removed if more than 30% of values were missing. If less than 30% of values were missing, feature imputation was performed using k-nearest neighbor imputation with distance weighting. However, only a small subset of features contained missing values and the imputed feature with the most missing values had only <14% missing values. To handle class imbalance, we employed the synthetic minority oversampling technique (SMOTE). Selection of features was performed using the minimum redundancy maximum relevance (mRMR) algorithm to select eight features (square root of the number of samples), reducing overfitting and redundancy. Hyperparameter optimization was conducted using random search through a predefined grid of reasonable parameters in a 10 x 5-fold nested cross-validation scheme. In the process of ML, the following packages were used:

- Graphviz 0.20
- Imbalanced-learn 0.8.0
- Numpy 1.25.2
- Pandas 1.4.2
- Pymrmr 0.1.11
- Scikit-learn 1.1.0
- Scipy 1.11.4
- Shap 0.44.1
- Umap-learn 0.5.3
- Xgboost 1.6.1
- Ydata-profiling 4.6.4

Supplementary Material—Results

R1: The radiomics profile based on permutation importance.

According to permutation importance, ten important imaging features (radiomics features and SUV metrics) contribute most to the prediction of whole mount Gleason grading in the ML model (**Supplementary Figure S6**). Among these features, texture features have the highest proportion, which accounts for 60% of the total observations. Only GLCM, GLDM, and GLRLM features contribute to the importance, of which GLCM features play the most important role. Histogram features are the next category with significance. Of note, maximum is the most important feature of all these features. For the conventional SUV metrics, SUVmean and SUVmax are important features for predicting whole mount Gleason grading.

A subgroup analysis of the key imaging features was conducted within each category, ranking them in detail (**Supplementary Figure S7**). For SUV metrics, four features out of 6 have permutation importance. For shape features, nine features out of 14 are essential. For histogram features, four features out of 18 are of vital importance. For texture features, 30 features out of 75 are crucial. The distribution of these essential features is shown in **Supplementary Figure S8.**

The interpretation of all radiomics features were respectively listed in **Supplementary Tables S5-7.**

R2: The pathomics profile based on permutation importance.

According to permutation importance, the five pathomic features, which are PSA, CD3, FASN, NKX3.1, STAT3 and CDK2, were identified as the most important features that contribute most to the ML model to predict the whole mount Gleason grading. Their importance values in ascending order were shown in **Supplementary Figure S9**.

R3: The ML performance after adding the additional MRI and fusion features and MRI-based scores.

We delineated the VOI on MRI and fusion images and derived the MRI- and fusion-based radiomics features. As part of our feature set, we have included established MRI scoring systems, such as the PI-RADS (Prostate Imaging-Reporting and Data System). This score provides a standardized assessment of lesion characteristics and has demonstrated clinical relevance and prognostic value. In this scenario, Prof. Pascal Baltzer and Prof. Thomas Helbich, radiologists with more than 20 years of experience in prostate cancer diagnosis, helped us assess the PI-RADS of our cohort. After inputting the MRI- and fusion-based radiomics features, the five ML model give the following performance. (**Supplementary Table S8**)

Supplementary Materials--Tables

Table S1. Data dictionary of all features and outcomes (labels) captured.

The number (percentage) of missing values for each feature and labels are provided.

**Features**	**Data type**	**Description of method of collection or measurement**	**Range of values for numerical features, coded values for categorical features**	**Missing values, n (%)**
**Clinical features**				
Age	Numerical	Age at the time of PET/MR examination, in years	42 to 75	0 (0)
Weight/kg	Numerical	Direct from clinical documentation	62 to 123	0 (0)
height/m	Numerical	Direct from clinical documentation	1.65 to 1.96	0 (0)
BMI	Numerical	Calculated based on the formular	20 to 36	0 (0)
PSA-pre OP µg/l	Numerical	Direct from laboratory documentation	1.95 to 827.8	0 (0)
Pre-op therapy	Binary	Direct from clinical documentation	0 = no; 1 = yes	2 (3.08%)
Lesion involvement	Categorical	Assessed by nuclear medicine physician based on PET	whether the tumor affected one or two lobes or was diffusely spread throughout the prostate;	22 (33.85%)

			1 = one lobe; 2 = two lobes; 3 = whole prostate	
Lesion position in anatomy zone	Categorical	Assessed by nuclear medicine physician based on PET	whether the tumor was located in the central zone (CZ), transition zone (TZ), peripheral zone (PZ), anterior fibromuscular stroma (AFS), or it was diffusely distributed (i.e., tumor lesions involving at least two anatomical zones or the whole prostate; 1 = central zone; 2 = transition zone; 3 = peripheral zone; 4 = anterior fibromuscular stroma; 5 = diffusion:	23 (35.38%)
Extracapsular extension	Binary	Assessed by nuclear medicine physician based on PET	whether the tumor exceeded the prostate capsule; 0 = no; 1 = yes	22 (33.85%)
Contact to neurovascular bundles	Binary	Assessed by nuclear medicine physician based on PET	whether the tumor infiltrated adjacent neurovascular bundles;	22 (33.85%)

			0 = no; 1 = yes	
Lymph nodes(LNs) infiltration	Binary	Assessed by nuclear medicine physician based on PET	whether the tumor infiltrated the pelvic or distant LNs; 0 = no; 1 = yes	21 (32.31%)
Bone metastasis	Binary	Assessed by nuclear medicine physician based on PET	whether tumor metastasized to bones; 0 = no; 1 = yes	22 (33.85%)
T staging PET	Categorical	Assessed by nuclear medicine physician based on PET	1 = cT2a; 2 = cT2b; 3 = cT2c; 4 = cT3a; 5 = cT3b; 6 = cT3a+b; 7 = cT4	20 (30.77%)
**Radiomics-wide features**				
SUVmin	Numerical	Computed from tumor VOI	0.44 to 9.79	0 (0)
SUVmax	Numerical	Computed from tumor VOI	3.39 to 73.05	0 (0)
SUVmean	Numerical	Computed from tumor VOI	1.89 to 27.22	0 (0)
SUVpeak	Numerical	Computed from tumor VOI	2.46 to 58.05	0 (0)
MTV	Numerical	Computed from tumor VOI	0.77 to 31.32	0 (0)
TLG	Numerical	Computed from tumor VOI	2.88 to 458.78	0 (0)
shape_Elongation	Numerical	Derived from tumor VOI on Pyradiomics	0.36 to 0.97	0 (0)

shape_Flatness	Numerical	Derived from tumor VOI on Pyradiomics	0.27 to 0.82	0 (0)
shape_Least Axis Length	Numerical	Derived from tumor VOI on Pyradiomics	7.76 to 32.20	0 (0)
shape_Major Axis Length	Numerical	Derived from tumor VOI on Pyradiomics	16.09 to 81.87	0 (0)
shape_Maximum 2D Diameter Column	Numerical	Derived from tumor VOI on Pyradiomics	12.0 to 70.46	0 (0)
shape_Maximum 2D Diameter Row	Numerical	Derived from tumor VOI on Pyradiomics	16.49 to 56.04	0 (0)
shape_Maximum 2D Diameter Slice	Numerical	Derived from tumor VOI on Pyradiomics	17.89 to 70.34	0 (0)
shape_Maximum 3D Diameter	Numerical	Derived from tumor VOI on Pyradiomics	20.20 to 71.16	0 (0)
shape_Mesh Volume	Numerical	Derived from tumor VOI on Pyradiomics	577.67 to 30049.67	0 (0)

shape_Minor Axis Length	Numerical	Derived from tumor VOI on Pyradiomics	8.87 to 42.59	0 (0)
shape_Sphericity	Numerical	Derived from tumor VOI on Pyradiomics	0.34 to 0.73	0 (0)
shape_Surface Area	Numerical	Derived from tumor VOI on Pyradiomics	500.55 to 12580.24	0 (0)
shape_Surface Volume Ratio	Numerical	Derived from tumor VOI on Pyradiomics	0.33 to 1.02	0 (0)
shape_Voxel Volume	Numerical	Derived from tumor VOI on Pyradiomics	640.0 to 30456.0	0 (0)
first order_10Percentile	Numerical	Derived from tumor VOI on Pyradiomics	1.30 to 17.75	0 (0)
first order_90Percentile	Numerical	Derived from tumor VOI on Pyradiomics	2.41 to 43.62	0 (0)
first order_Energy	Numerical	Derived from tumor VOI on Pyradiomics	891.73 to 1855871.76	0 (0)

first order_Entropy	Numerical	Derived from tumor VOI on Pyradiomics	1.88 to 7.22	0 (0)
first order_Interquartile Range	Numerical	Derived from tumor VOI on Pyradiomics	0.37 to 15.60	0 (0)
first order_Kurtosis	Numerical	Derived from tumor VOI on Pyradiomics	1.92 to 9.03	0 (0)
first order_Maximum	Numerical	Derived from tumor VOI on Pyradiomics	3.34 to 72.95	0 (0)
first order_Mean Absolute Deviation	Numerical	Derived from tumor VOI on Pyradiomics	0.24 to 9.65	0 (0)
first order_Mean	Numerical	Derived from tumor VOI on Pyradiomics	1.90 to 26.99	0 (0)
first order_Median	Numerical	Derived from tumor VOI on Pyradiomics	1.88 to 26.08	0 (0)
first order_Minimum	Numerical	Derived from tumor VOI on Pyradiomics	0.39 to 8.45	0 (0)

first order_Range	Numerical	Derived from tumor VOI on Pyradiomics	1.24 to 70.03	0 (0)
first order_Robust Mean Absolute Deviation	Numerical	Derived from tumor VOI on Pyradiomics	0.17 to 6.57	0 (0)
first order_Root Mean Squared	Numerical	Derived from tumor VOI on Pyradiomics	1.94 to 29.65	0 (0)
firstorder_Skewness	Numerical	Derived from tumor VOI on Pyradiomics	-0.72 to 2.15	0 (0)
first order_Total Energy	Numerical	Derived from tumor VOI on Pyradiomics	7133.83 to 14846974.06	0 (0)
first order_Uniformity	Numerical	Derived from tumor VOI on Pyradiomics	0.01 to 0.31	0 (0)
first order_Variance	Numerical	Derived from tumor VOI on Pyradiomics	0.09 to 150.63	0 (0)
GLCM_Autocorrelation	Numerical	Derived from tumor VOI on Pyradiomics	18.83 to 9363.16	0 (0)

GLCM_Cluster Prominence	Numerical	Derived from tumor VOI on Pyradiomics	10.69 to 115837280.1	0 (0)
GLCM_Cluster Shade	Numerical	Derived from tumor VOI on Pyradiomics	1922.90 to 298080.79	0 (0)
GLCM_Cluster Tendency	Numerical	Derived from tumor VOI on Pyradiomics	1.69 to 5842.78	0 (0)
GLCM_Contrast	Numerical	Derived from tumor VOI on Pyradiomics	0.72 to 782.93	0 (0)
GLCM_Correlation	Numerical	Derived from tumor VOI on Pyradiomics	0.38 to 0.95	0 (0)
GLCM_Difference Average	Numerical	Derived from tumor VOI on Pyradiomics	0.59 to 21.79	0 (0)
GLCM_Difference Entropy	Numerical	Derived from tumor VOI on Pyradiomics	1.21 to 5.76	0 (0)
GLCM_Difference Variance	Numerical	Derived from tumor VOI on Pyradiomics	0.35 to 289.49	0 (0)

GLCM_Id	Numerical	Derived from tumor VOI on Pyradiomics	0.11 to 0.73	0 (0)
GLCM_Idm	Numerical	Derived from tumor VOI on Pyradiomics	0.05 to 0.72	0 (0)
GLCM_Idmn	Numerical	Derived from tumor VOI on Pyradiomics	0.96 to 0.10	0 (0)
GLCM_Idn	Numerical	Derived from tumor VOI on Pyradiomics	0.87 to 0.97	0 (0)
GLCM_Imc1	Numerical	Derived from tumor VOI on Pyradiomics	-0.56 to -0.13	0 (0)
GLCM_Imc2	Numerical	Derived from tumor VOI on Pyradiomics	0.59 to 0.10	0 (0)
GLCM_Inverse Variance	Numerical	Derived from tumor VOI on Pyradiomics	0.05 to 0.50	0 (0)
GLCM_Joint Average	Numerical	Derived from tumor VOI on Pyradiomics	4.25 to 89.97	0 (0)

GLCM_Joint Energy	Numerical	Derived from tumor VOI on Pyradiomics	0.00 to 0.19	0 (0)
GLCM_Joint Entropy	Numerical	Derived from tumor VOI on Pyradiomics	2.76 to 11.35	0 (0)
GLCM_MCC	Numerical	Derived from tumor VOI on Pyradiomics	0.49 to 0.10	0 (0)
GLCM_Maximum Probability	Numerical	Derived from tumor VOI on Pyradiomics	0.00 to 0.29	0 (0)
GLCM_Sum Average	Numerical	Derived from tumor VOI on Pyradiomics	8.50 to 179.93	0 (0)
GLCM_Sum Entropy	Numerical	Derived from tumor VOI on Pyradiomics	2.11 to 8.02	0 (0)
GLCM_Sum Squares	Numerical	Derived from tumor VOI on Pyradiomics	0.60 to 1656.43	0 (0)
GLRLM_Gray Level Non Uniformity	Numerical	Derived from tumor VOI on Pyradiomics	7.74 tto 263.04	0 (0)

GLRLM_Gray Level Non Uniformity Normalized	Numerical	Derived from tumor VOI on Pyradiomics	0.01 to 0.30	0 (0)
GLRLM_Gray Level Variance	Numerical	Derived from tumor VOI on Pyradiomics	1.09 to 1679.98	0 (0)
GLRLM_High Gray Level Run Emphasis	Numerical	Derived from tumor VOI on Pyradiomics	15.48 to 8316.15	0 (0)
GLRLM_Long Run Emphasis	Numerical	Derived from tumor VOI on Pyradiomics	1.04 to 2.44	0 (0)
GLRLM_Long Run High Gray Level Emphasis	Numerical	Derived from tumor VOI on Pyradiomics	29.03 to 8611.91	0 (0)
GLRLM_Long Run Low Gray Level Emphasis	Numerical	Derived from tumor VOI on Pyradiomics	0.00 to 0.21	0 (0)
GLRLM_Low Gray Level Run Emphasis	Numerical	Derived from tumor VOI on Pyradiomics	0.00 to 0.13	0 (0)

GLRLM_Run Entropy	Numerical	Derived from tumor VOI on Pyradiomics	2.64 to 7.29	0 (0)
GLRLM_Run Length Non Uniformity	Numerical	Derived from tumor VOI on Pyradiomics	38.39 to 3206.36	0 (0)
GLRLM_Run Length Non Uniformity Normalized	Numerical	Derived from tumor VOI on Pyradiomics	0.61 to 0.98	0 (0)
GLRLM_Run Percentage	Numerical	Derived from tumor VOI on Pyradiomics	0.76 to 0.99	0 (0)
GLRLM_Run Variance	Numerical	Derived from tumor VOI on Pyradiomics	0.01 to 0.61	0 (0)
GLRLM_Short Run Emphasis	Numerical	Derived from tumor VOI on Pyradiomics	0.79 to 0.99	0 (0)
GLRLM_Short Run High Gray Level Emphasis	Numerical	Derived from tumor VOI on Pyradiomics	11.22 to 8245.42	0 (0)

GLRLM_Short Run Low Gray Level Emphasis	Numerical	Derived from tumor VOI on Pyradiomics	0.00 to 0.11	0 (0)
GLSZM_Gray Level Non Uniformity	Numerical	Derived from tumor VOI on Pyradiomics	2.83 to 18595.0	0 (0)
GLSZM_Gra yLeve lNon Uniformity Normalized	Numerical	Derived from tumor VOI on Pyradiomics	0.01 to 0.24	0 (0)
GLSZM_Gray Level Variance	Numerical	Derived from tumor VOI on Pyradiomics	1.39 to 1757.813	0 (0)
GLSZM_High Gray Level Zone Emphasis	Numerical	Derived from tumor VOI on Pyradiomics	8.5 to 8394.619154	0 (0)
GLSZM_Large Area Emphasis	Numerical	Derived from tumor VOI on Pyradiomics	1.63 to 55875.0	0 (0)
GLSZM_Large Area High Gray Level Emphasis	Numerical	Derived from tumor VOI on Pyradiomics	965.03 to 229276.03	0 (0)

GLSZM_Large Area Low Gray Level Emphasis	Numerical	Derived from tumor VOI on Pyradiomics	0.00 to 425.09	0 (0)
GLSZM_Low Gray Level Zone Emphasis	Numerical	Derived from tumor VOI on Pyradiomics	0.00 to 0.29	0 (0)
GLSZM_Size Zone Non Uniformity	Numerical	Derived from tumor VOI on Pyradiomics	3.67 to 1362.06	0 (0)
GLSZM_Size Zone Non Uniformity Normalized	Numerical	Derived from tumor VOI on Pyradiomics	0.10 to 0.67	0 (0)
GLSZM_Small Area Emphasis	Numerical	Derived from tumor VOI on Pyradiomics	0.25 to 0.89	0 (0)
GLSZM_Small Area High Gray Level Emphasis	Numerical	Derived from tumor VOI on Pyradiomics	2.52 to 7576.37	0 (0)

GLSZM_Small Area Low Gray Level Emphasis	Numerical	Derived from tumor VOI on Pyradiomics	0.00 to 2.24	0 (0)
GLSZM_Zone Entropy	Numerical	Derived from tumor VOI on Pyradiomics	2.92 to 7.81	0 (0)
GLSZM_Zone Percentage	Numerical	Derived from tumor VOI on Pyradiomics	0.03 to 0.85	0 (0)
GLSZM_Zone Variance	Numerical	Derived from tumor VOI on Pyradiomics	0.24 to 8038.92	0 (0)
GLDM_Dependence Entropy	Numerical	Derived from tumor VOI on Pyradiomics	4.44 to 8.04	0 (0)
GLDM_Dependence Non Uniformity	Numerical	Derived from tumor VOI on Pyradiomics	29.62 to 7175.0	0 (0)
GLDM_Dependence Non Uniformity Normalized	Numerical	Derived from tumor VOI on Pyradiomics	0.08 to 0.59	0 (0)

GLDM_Dependence Variance	Numerical	Derived from tumor VOI on Pyradiomics	0.33 to 26.67	0 (0)
GLDM_Gray Level Non Uniformity	Numerical	Derived from tumor VOI on Pyradiomics	8.01 to 362.96	0 (0)
GLDM_Gray Level Variance	Numerical	Derived from tumor VOI on Pyradiomics	1.02 to 1673.89	0 (0)
GLDM_High Gray Level Emphasis	Numerical	Derived from tumor VOI on Pyradiomics	16.60 to 16825.00	0 (0)
GLDM_Large Dependence Emphasis	Numerical	Derived from tumor VOI on Pyradiomics	2.06 to 69.2	0 (0)
GLDM_Large Dependence High Gray Level Emphasis	Numerical	Derived from tumor VOI on Pyradiomics	489.26 to 18943.31	0 (0)
GLDM_Large Dependence Low Gray Level Emphasis	Numerical	Derived from tumor VOI on Pyradiomics	0.00 to 23.56	0 (0)

GLDM_Low Gray Level Emphasis	Numerical	Derived from tumor VOI on Pyradiomics	0.00 to 0.12	0 (0)
GLDM_Small Dependence Emphasis	Numerical	Derived from tumor VOI on Pyradiomics	0.04 to 0.79	0 (0)
GLDM_Small Dependence High Gray Level Emphasis	Numerical	Derived from tumor VOI on Pyradiomics	0.84 to 6714.97	0 (0)
GLDM_Small Dependence Low Gray Level Emphasis	Numerical	Derived from tumor VOI on Pyradiomics	0.00 to 0.04	0 (0)
NGTDM_Busyness	Numerical	Derived from tumor VOI on Pyradiomics	0.01 to 0.49	0 (0)
NGTDM_Coarseness	Numerical	Derived from tumor VOI on Pyradiomics	0.00 to 0.10	0 (0)
NGTDM_Complexity	Numerical	Derived from tumor VOI on Pyradiomics	4.74 to 188223.78	0 (0)

NGTDM_Contrast	Numerical	Derived from tumor VOI on Pyradiomics	0.02 to 0.82	0 (0)
NGTDM_Strength	Numerical	Derived from tumor VOI on Pyradiomics	0.42 to 146.63	0 (0)
**Genomic features**				
Citrate cycle (TCA cycle)	Numerical	Combined pathogecity scores by bioinformatics analysis	0 to 2.51	0 (0)
Fatty acid biosynthesis	Numerical	Combined pathogecity scores by bioinformatics analysis	0 to 3.47	0 (0)
Fatty acid elongation	Numerical	Combined pathogecity scores by bioinformatics analysis	0 to 5.74	0 (0)
Fatty acid degradation	Numerical	Combined pathogecity scores by bioinformatics analysis	0 to 3.33	0 (0)
Cysteine and methionine metabolism	Numerical	Combined pathogecity scores by bioinformatics analysis	0 to 3.60	0 (0)

One carbon pool by folate	Numerical	Combined pathogecity scores by bioinformatics analysis	0 to 3.39	0 (0)
Folate biosynthesis	Numerical	Combined pathogecity scores by bioinformatics analysis	0 to 1.26	0 (0)
Metabolic pathways	Numerical	Combined pathogecity scores by bioinformatics analysis	0 to 108.27	0 (0)
Fatty acid metabolism	Numerical	Combined pathogecity scores by bioinformatics analysis	0 to 5.80	0 (0)
EGFR tyrosine kinase inhibitor resistance	Numerical	Combined pathogecity scores by bioinformatics analysis	0 to 14.07	0 (0)
Antifolate resistance	Numerical	Combined pathogecity scores by bioinformatics analysis	0 to 4.76	0 (0)
PPAR signaling pathway	Numerical	Combined pathogecity scores by bioinformatics analysis	0 to 5.92	0 (0)
MAPK signaling pathway	Numerical	Combined pathogecity scores by bioinformatics analysis	0 to 29.03	0 (0)

Ras signaling pathway	Numerical	Combined pathogecity scores by bioinformatics analysis	0 to 17.92	0 (0)
Rap1 signaling pathway	Numerical	Combined pathogecity scores by bioinformatics analysis	0 to 20.62	0 (0)
Calcium signaling pathway	Numerical	Combined pathogecity scores by bioinformatics analysis	0 to 23.51	0 (0)
cAMP signaling pathway	Numerical	Combined pathogecity scores by bioinformatics analysis	0 to 15.97	0 (0)
NF-kappa B signaling pathway	Numerical	Combined pathogecity scores by bioinformatics analysis	0 to 9.36	0 (0)
HIF-1 signaling pathway	Numerical	Combined pathogecity scores by bioinformatics analysis	0 to 11.50	0 (0)
FoxO signaling pathway	Numerical	Combined pathogecity scores by bioinformatics analysis	0 to 11.68	0 (0)
Cell cycle	Numerical	Combined pathogecity scores by bioinformatics analysis	0 to 7.16	0 (0)

p53 signaling pathway	Numerical	Combined pathogecity scores by bioinformatics analysis	0 to 3.99	0 (0)
mTOR signaling pathway	Numerical	Combined pathogecity scores by bioinformatics analysis	0 to 22.45	0 (0)
PI3K-Akt signaling pathway	Numerical	Combined pathogecity scores by bioinformatics analysis	0 to 37.22	0 (0)
Apoptosis	Numerical	Combined pathogecity scores by bioinformatics analysis	0 to 17.36	0 (0)
Cellular senescence	Numerical	Combined pathogecity scores by bioinformatics analysis	0 to 25.75	0 (0)
Wnt signaling pathway	Numerical	Combined pathogecity scores by bioinformatics analysis	0 to 18.24	0 (0)
Notch signaling pathway	Numerical	Combined pathogecity scores by bioinformatics analysis	0 to 7.25	0 (0)
Hedgehog signaling pathway	Numerical	Combined pathogecity scores by bioinformatics analysis	0 to 5.36	0 (0)

TGF-beta signaling pathway	Numerical	Combined pathogecity scores by bioinformatics analysis	0 to 4.58	0 (0)
VEGF signaling pathway	Numerical	Combined pathogecity scores by bioinformatics analysis	0 to 6.12	0 (0)
Focal adhesion	Numerical	Combined pathogecity scores by bioinformatics analysis	0 to 29.85	0 (0)
ECM-receptor interaction	Numerical	Combined pathogecity scores by bioinformatics analysis	0 to 16.26	0 (0)
Adherens junction	Numerical	Combined pathogecity scores by bioinformatics analysis	0 to 10.17	0 (0)
Neutrophil extracellular trap formation	Numerical	Combined pathogecity scores by bioinformatics analysis	0 to 15.38	0 (0)
Toll-like receptor signaling pathway	Numerical	Combined pathogecity scores by bioinformatics analysis	0 to 10.82	0 (0)
JAK-STAT signaling pathway	Numerical	Combined pathogecity scores by bioinformatics analysis	0 to 16.82	0 (0)

Natural killer cell mediated cytotoxicity	Numerical	Combined pathogecity scores by bioinformatics analysis	0 to 58.01	0 (0)
TNF signaling pathway	Numerical	Combined pathogecity scores by bioinformatics analysis	0 to 9.41	0 (0)
Insulin signaling pathway	Numerical	Combined pathogecity scores by bioinformatics analysis	0 to 17.59	0 (0)
Thyroid hormone synthesis	Numerical	Combined pathogecity scores by bioinformatics analysis	0 to 5.32	0 (0)
Thyroid hormone signaling pathway	Numerical	Combined pathogecity scores by bioinformatics analysis	0 to 18.28	0 (0)
Endocrine and other factor-regulated calcium reabsorption	Numerical	Combined pathogecity scores by bioinformatics analysis	0 to 4.84	0 (0)
Pathways of neurodegeneration - multiple diseases	Numerical	Combined pathogecity scores by bioinformatics analysis	0 to 40.80	0 (0)

Human immunodeficiency virus 1 infection	Numerical	Combined pathogecity scores by bioinformatics analysis	0 to 24.84	0 (0)
Pathways in cancer	Numerical	Combined pathogecity scores by bioinformatics analysis	0 to 56.59	0 (0)
Glioma	Numerical	Combined pathogecity scores by bioinformatics analysis	0 to 8.68	0 (0)
Prostate cancer	Numerical	Combined pathogecity scores by bioinformatics analysis	0 to 13.19	0 (0)
Thyroid cancer	Numerical	Combined pathogecity scores by bioinformatics analysis	0 to 3.77	0 (0)
Choline metabolism in cancer	Numerical	Combined pathogecity scores by bioinformatics analysis	0 to 14.12	0 (0)
PD-L1 expression and PD-1 checkpoint pathway in cancer	Numerical	Combined pathogecity scores by bioinformatics analysis	0 to 10.73	0 (0)

Tumor mutational burden (TMB)	Numerical	computed as a number of identified somatic variants per million base pairs of the sequence region	1.24 to 39.33	0 (0)
Copy number variant (CNV) burden	Numerical	Computed as a ratio of CNV sum size to the sum size of all sequenced regions	0 to 18.7	9 (13.85%)
**ISUP in needle biopsy**	Categorical	The maximum H-score among all tumor cores	1 to 5	0 (0)
**Pathomics features**				
Ki-67max	Numerical	The maximum H-score of Ki-67 staining among all tumor cores	0 to 130	1 (1.54%)
Ki-67avg	Numerical	The average H-score of Ki-67 staining among all tumor cores	0 to 108.33	1 (1.54%)
PSMAmax	Numerical	The maximum H-score of PSMA staining among all tumor cores	0 to 300	1 (1.54%)
PSMAavg	Numerical	The average H-score of PSMA staining among all tumor cores	0 to 300	1 (1.54%)

ARmax	Numerical	The maximum H-score of AR staining among all tumor cores	0 to 300	1 (1.54%)
ARavg	Numerical	The average H-score of AR staining among all tumor cores	0 to 300	1 (1.54%)
PSAmax	Numerical	The maximum H-score of PSA staining among all tumor cores	0 to 300	5 (7.69%)
PSAavg	Numerical	The average H-score of PSA staining among all tumor cores	0 to 200	5 (7.69%)
NKX3.1max	Numerical	The maximum H-score of NKX3.1 staining among all tumor cores	0 to 300	1 (1.54%)
NKX3.1avg	Numerical	The average H-score of NKX3.1 staining among all tumor cores	0 to 260	1 (1.54%)
CDK2max	Numerical	The maximum H-score of CDK2 staining among all tumor cores	0 to 36	1 (1.54%)
CDK2avg	Numerical	The average H-score of CDK2 staining among all tumor cores	0 to 27	1 (1.54%)

STAT3max	Numerical	The maximum H-score of STAT3 staining among all tumor cores	0 to 180	2 (3.08%)
STAT3avg	Numerical	The average H-score of STAT3 staining among all tumor cores	0 to 85	2 (3.08%)
CD3max	Numerical	The maximum H-score of CD3 staining among all tumor cores	0 to 40	1 (1.54%)
CD3avg	Numerical	The average H-score of CD3 staining among all tumor cores	0 to 20	1 (1.54%)
FASNmax	Numerical	The maximum H-score of FASN staining among all tumor cores	0 to 300	0 (0)
FASNavg	Numerical	The average H-score of FASN staining among all tumor cores	0 to 250	0 (0)
TRβmax	Numerical	The maximum H-score of TRβ staining among all tumor cores	0 to 300	1 (1.54%)
Trβavg	Numerical	The average H-score of TRβ staining among all tumor cores	0 to 250	1 (1.54%)

IL6ST	Categorical	Derived from tumor cores stained with IL6ST	0 = no IL6ST expression; 1 = low IL6ST expression; 2 = high IL6ST expression	1 (1.54%)
infiltration to tumor	Categorical	Derived from tumor cores stained with IL6ST	0 = no; 1 = yes	1 (1.54%)
infiltration to normal	Categorical	Derived from tumor cores stained with IL6ST	0 = no; 1 = yes	1 (1.54%)

Notes: Regarding radiomics features, PET images were resampled to an isotropic voxel size of 2x2x2 mm^3 using B-spline interpolation and bin width was set to 0.3 SUV units.

Table S2. The investigated 51 pathways and matched literature.

A total of 51 categorized pathways and the corresponding literature is provided to indicate their role in PCa tumorigenesis, progression and metastasis.

Feature roup	Pathways
**PSMA-related pathways**	Antifolate resistance[17]<br>One carbon pool by folate[17]<br>Folate biosynthesis[17]<br>Endocrine and other factor-regulated calcium reabsorption[17]<br>Calcium signaling pathway[17]<br>Cysteine and methionine metabolism[18]<br>PI3K-Akt signaling pathway[19]<br>MAPK signaling pathway[19, 20]<br>VEGF signaling pathway[20, 21]
**Anrogen receptor-related pathways**	Glioma[22]<br>Pathways of neurodegeneration - multiple diseases[23, 24]<br>Prostate cancer[25]
**General pathways known for Pca**	Wnt signaling pathway[20]<br>mTOR signaling pathway[20]<br>EGFR tyrosine kinase inhibitor resistance[20]<br>TGF-beta signaling pathway[20]<br>NF-kappa B signaling pathway[25]<br>JAK-STAT signaling pathway[25]<br>Ras signaling pathway[25]<br>Pathways in cancer[25]<br>Hedgehog signaling pathway[26]<br>Notch signaling pathway[26]

	FoxO signaling pathway[26]<br>Adherens junction[27]<br>Cellular senescence[28]<br>Cell cycle[29]<br>TNF signaling pathway[30]<br>p53 signaling pathway[30]<br>HIF-1 signaling pathway[31]<br>PPAR signaling pathway[32]<br>Apoptosis[33]
**Metabolism-related pathways**	Choline metabolism in cancer[34]<br>Fatty acid metabolism[35]<br>Fatty acid biosynthesis[35, 36]<br>Fatty acid degradation[35, 36]<br>Fatty acid elongation[35, 36]<br>Citrate cycle (TCA cycle)[37, 38]<br>Metabolic pathways [37, 38]<br>Insulin signaling pathway[39]
**Membrane recruitment and activation**	ECM-receptor interaction[40]<br>Focal adhesion[41]<br>cAMP signaling pathway[42]<br>Rap1 signaling pathway[43]
**Thyroid connection**	Thyroid cancer[44]<br>Thyroid hormone signaling pathway[44]<br>Thyroid hormone synthesis[44]
**Immunology-related pathways**	PD-L1 expression and PD-1 checkpoint pathway in cancer[45]<br>Neutrophil extracellular trap formation[46]<br>Human immunodeficiency virus 1 infection[47]

	Toll-like receptor signaling pathway[48]<br><br>Natural killer cell mediated cytotoxicity[49]

Table S3. The extracted radiomics features in three categories.

The extracted radiomic features from ^{68}Ga-PSMA PET/MR images: Shape-based features including 14 shape dimentions; First-order features including 18 intensity statistics; 75 multi-dimensional texture features including 24 Gray Level Co-occurrence Matrix (GLCM), 16 Gray Level Run Length Matrix (GLRLM),16 Gray Level Size Zone Matrix (GLSZM), 14 Gray Level Dependence Matrix (GLDM) and 5 Neighboring Gray Tone Difference Matrix (NGTDM) Features.

**Feature group**	**Feature name**
Shape-based (n=14)	Elongation
	Flatness
	Least Axis Length
	Major Axis Length
	Maximum2D Diameter Column
	Maximum2D Diameter Row
	Maximum2D Diameter Slice
	Maximum3D Diameter
	MeshVolume
	MinorAxisLength
	Sphericity
	Surface Area
	Surface Volume Ratio
	Voxel Volume
Histogram-based (n=18)	10Percentile
	90Percentile
	Energy

	Entropy	
	Interquartile Range	
	Kurtosis	
	Maximum	
	Mean Absolute Deviation	
	Mean	
	Median	
	Minimum	
	Range	
	Robust Mean Absolute Deviation	
	Root Mean Squared	
	Skewness	
	Total Energy	
	Uniformity	
	Variance	
Texture-based (n=75)	GLCM (n=24)	Autocorrelation
		Cluster Prominence
		Cluster Shade
		Cluster Tendency
		Contrast
		Correlation
		Difference Average
		Difference Entropy
		Difference Variance
		Id
		Idm
		Idmn

		Idn
		Imc1
		Imc2
		Inverse Variance
		Joint Average
		Joint Energy
		Joint Entropy
		MCC
		Maximum Probability
		Sum Average
		Sum Entropy
		Sum Squares
	GLRLM (n=16)	Gray Level Non Uniformity
		Gray Level Non Uniformity Normalized
		Gray Level Variance
		High Gray Level Run Emphasis
		Long Run Emphasis
		Long Run High Gray Level Emphasis
		Long Run Low Gray Level Emphasis
		Low Gray Level Run Emphasis
		Run Entropy
		Run Length Non Uniformity
		Run Length Non Uniformity Normalized
		Run Percentage
		Run Variance
		Short Run Emphasis
		Short Run High Gray Level Emphasis

		Short Run Low Gray Level Emphasis
	GLSZM (n=16)	Gray Level Non Uniformity
		Gray Level Non Uniformity Normalized
		Gray Level Variance
		High Gray Level Zone Emphasis
		Large Area Emphasis
		Large Area High Gray Level Emphasis
		Large Area Low Gray Level Emphasis
		Low Gray Level Zone Emphasis
		Size Zone Non Uniformity
		Size Zone Non Uniformity Normalized
		Small Area Emphasis
		Small Area High Gray Level Emphasis
		Small Area Low Gray Level Emphasis
		Zone Entropy
		Zone Percentage
		Zone Variance
	GLDM (n=16)	Dependence Entropy
		Dependence Non Uniformity
		Dependence Non Uniformity Normalized
		Dependence Variance
		Gray Level Non Uniformity
		Gray Level Variance
		High Gray Level Emphasis
		Large Dependence Emphasis
		Large Dependence High Gray Level Emphasis

		Large Dependence Low Gray Level Emphasis
		Low Gray Level Emphasis
		Small Dependence Emphasis
		Small Dependence High Gray Level Emphasis
		Small Dependence Low Gray Level Emphasis
	NGTDM (n=5)	Busyness
		Coarseness
		Complexity
		Contrast
		Strength

Table S4. The performance metrics of five different ML models to predict Gleason grading in PCa.

	**ACC**	**SNS**	**SPC**	**PPV**	**NPV**	**AUC**
**KNN**	0.754	0.766	0.740	0.791	0.754	0.828
**RF**	0.779	0.827	0.722	0.791	0.804	0.869
**SVM**	0.757	0.816	0.688	0.770	0.768	0.853
**LGR**	0.748	0.761	0.732	0.788	0.742	0.835
**XGB**	0.770	0.831	0.698	0.778	0.803	0.868

AUC: area under the curve; SNS: sensitivity; SPC: specificity; ACC: accuracy; PPV: positive predictive value; NPV: negative predictive value; KNN: K-nearest neighbors; RF: random forest; SVM: support vector machines; IGR: information gain ratio; XGB: extreme gradient boosting.

Table S*5*. The interpretation of shape-based features ranked by descending contribution to the prediction of whole mount Gleason grading in the ML model.

Feature Name	Formula	Meaning
Maximum 2D diameter (Slice)	NA	Maximum 2D diameter (Slice) is defined as the largest pairwise Euclidean distance between tumor surface mesh vertices in the row-column (generally the axial) plane.
Maximum 3D diameter	NA	Maximum 3D diameter is defined as the largest pairwise Euclidean distance between tumor surface mesh vertices. Also known as Feret Diameter.
Elongation	$elongation = \sqrt{\frac{\lambda_{minor}}{\lambda_{major}}}$ Here, λmajor and λminor are the lengths of the largest and second largest principal component axes. The values range between 1 (where the cross section through the first and second largest principal moments is circle-like (non-elongated)) and 0 (where the object is a maximally elongated: i.e. a 1 dimensional line).	Elongation shows the relationship between the two largest principal components in the ROI shape. For computational reasons, this feature is defined as the inverse of true elongation.<br><br>The principal component analysis is performed using the physical coordinates of the voxel centers defining the ROI. It therefore takes spacing into account, but does not make use of the shape mesh.

Maximum 2D diameter (Column)	NA	Maximum 2D diameter (Column) is defined as the largest pairwise Euclidean distance between tumor surface mesh vertices in the row-slice (usually the coronal) plane.
Major Axis Length	$major\ axis = 4\sqrt{\lambda_{major}}$	This feature yield the largest axis length of the ROI-enclosing ellipsoid and is calculated using the largest principal component λ_{major}.The principal component analysis is performed using the physical coordinates of the voxel centers defining the ROI. It therefore takes spacing into account, but does not make use of the shape mesh.
Least Axis Length	$least\ axis = 4\sqrt{\lambda_{least}}$	This feature yield the smallest axis length of the ROI-enclosing ellipsoid and is calculated using the largest principal component λ_{least}. In case of a 2D segmentation, this value will be 0. The principal component analysis is performed using the physical coordinates of the voxel centers defining the ROI. It therefore takes spacing into account, but does not make use of the shape mesh.
Flatness	$flatness = \sqrt{\frac{\lambda_{least}}{\lambda_{major}}}$ Here, λmajor and λleast are the lengths of the largest and smallest principal component	Flatness shows the relationship between the largest and smallest principal components in the ROI shape. For computational reasons, this feature is defined as the inverse of true flatness. The principal component analysis is performed

	axes. The values range between 1 (non-flat, sphere-like) and 0 (a flat object, or single-slice segmentation).	using the physical coordinates of the voxel centersdefining the ROI. It therefore takes spacing into account, but does not make use of the shape mesh.
Sphericity	$sphericity = \frac{\sqrt[3]{36\pi V^2}}{A}$	Sphericity is a measure of the roundness of the shape of the tumor region relative to a sphere. It is a dimensionless measure, independent of scale and orientation. The value range is $0 < sphericity \leq 1$, where a value of 1 indicates a perfect sphere (a sphere has the smallest possible surface area for a given volume, compared to other solids).
Surface Area	$A_i = \frac{1}{2}\lvert \mathbf{a}_i\mathbf{b}_i \times \mathbf{a}_i\mathbf{c}_i \rvert$ (1) $A = \sum_{i=1}^{N_f} A_i$ (2) where: a_ib_i and a_ic_i are edges of the ith triangle in the mesh, formed by vertices a_i, b_i and c_i.	To calculate the surface area, first the surface area A_i of each triangle in the mesh is calculated (1). The total surface area is then obtained by taking the sum of all calculated subareas.

Table S*6*. The interpretation of histogram-based features ranked by descending contribution to the prediction of whole mount Gleason grading in the ML model.

Feature Name	Formula	Meaning
Maximum	$maximum = \max(\mathbf{X})$	The maximum gray level intensity within the ROI.
Entropy	$entropy = -\sum_{i=1}^{N_g} p(i) \log_2 \left(p(i) + \epsilon\right)$ Here, ϵ is an arbitrarily small positive number ($\approx 2.2 \times 10^{-16}$).	Entropy specifies the uncertainty/randomness in the image values. It measures the average amount of information required to encode the image values.
Range	$range = \max(\mathbf{X}) - \min(\mathbf{X})$	The range of gray values in the ROI.
Minimum	$minimum = \min(\mathbf{X})$	NA

Table S7. The interpretation of texture-based features ranked by descending contribution to the prediction of whole mount Gleason grading in the ML model.

Class	Feature	Formula	Meaning
GLCM	Joint Entropy	$joint\ entropy = -\sum_{i=1}^{N_g}\sum_{j=1}^{N_g} p(i,j)\log_2\big(p(i,j)+\epsilon\big)$	Joint entropy is a measure of the randomness/variability in neighborhood intensity values.
GLRLM	Short Run Emphasis	$SRE = \frac{\sum_{i=1}^{N_g}\sum_{j=1}^{N_r}\frac{\mathbf{P}(i,j\|\theta)}{j^2}}{N_r(\theta)}$	SRE is a measure of the distribution of short run lengths, with a greater value indicative of shorter run lengths and more fine textural textures.
GLDM	Large Dependence Low Gray Level Emphasis	$LDLGLE = \frac{\sum_{i=1}^{N_g}\sum_{j=1}^{N_d}\frac{\mathbf{P}(i,j)j^2}{i^2}}{N_z}$	Measures the joint distribution of large Dependence with lower gray-level values
GLCM	Maximum Probability	$maximum\ probability = \max\big(p(i,j)\big)$	Maximum Probability is occurrences of the most predominant pair of neighboring intensity values
GLCM	Joint Average	$joint\ average = \mu_x = \sum_{i=1}^{N_g}\sum_{j=1}^{N_g} p(i,j)i$	Returns the mean gray level intensity of the i distribution.
GLCM	Difference Entropy	$difference\ entropy = \sum_{k=0}^{N_g-1} p_{x-y}(k)\log_2\big(p_{x-y}(k)+\epsilon\big)$	Difference Entropy is a measure of the randomness/variability in

			neighborhood intensity value differences.
GLRLM	Run Variance	$RV = \sum_{i=1}^{N_g}\sum_{j=1}^{N_r} p(i,j\|\theta)(j-\mu)^2$	RV is a measure of the variance in runs for the run lengths
GLCM	Sum Average	$sum\ average = \sum_{k=2}^{2N_g} p_{x+y}(k)k$	Sum Average measures the relationship between occurrences of pairs with lower intensity values and occurrences of pairs with higher intensity values.
GLSZM	Size Zone Non Uniformity	$SZN = \frac{\sum_{j=1}^{N_s}\left(\sum_{i=1}^{N_g}\mathbf{P}(i,j)\right)^2}{N_z}$	SZN measures the variability of size zone volumes in the image, with a lower value indicating more homogeneity in size zone volumes.
GLCM	Joint Energy	$joint\ energy = \sum_{i=1}^{N_g}\sum_{j=1}^{N_g}\left(p(i,j)\right)^2$	Energy is a measure of homogeneous patterns in the image. A greater Energy implies that there are more instances of intensity value pairs in the image that neighbor each other at higher frequencies.

GLCM	Sum Entropy	$$\textit{sum entropy} = \sum_{k=2}^{2N_g} p_{x+y}(k) \log_2 \left(p_{x+y}(k) + \epsilon\right)$$	Sum Entropy is a sum of neighborhood intensity value differences.
NGTDM	Busyness	$$Busyness = \frac{\sum_{i=1}^{N_g} p_i s_i}{\sum_{i=1}^{N_g} \sum_{j=1}^{N_g} \lvert ip_i - jp_j \rvert}, \text{ where } p_i \neq 0, p_j \neq 0$$	A measure of the change from a pixel to its neighbour. A high value for busyness indicates a 'busy' image, with rapid changes of intensity between pixels and its neighbourhood.
GLCM	Difference Average	$$\textit{difference average} = \sum_{k=0}^{N_g - 1} k p_{x-y}(k)$$	Difference Average measures the relationship between occurrences of pairs with similar intensity values and occurrences of pairs with differing intensity values.
GLSZM	Zone Percentage	$$ZP = \frac{N_z}{N_p}$$	ZP measures the coarseness of the texture by taking the ratio of number of zones and number of voxels in the ROI.
GLSZM	Size Zone Non Uniformity Normalized	$$SZNN = \frac{\sum_{j=1}^{N_s} \left(\sum_{i=1}^{N_g} \mathbf{P}(i,j)\right)^2}{N_z^2}$$	SZNN measures the variability of size zone volumes throughout the image, with a lower value indicating more homogeneity among zone size volumes in the image. This is the

			normalized version of the SZN formula.
GLRLM	Gray Level Non Uniformity	$GLN = \frac{\sum_{i=1}^{N_g} \left(\sum_{j=1}^{N_r} \mathbf{P}(i,j\|\theta) \right)^2}{N_r(\theta)}$	GLN measures the similarity of gray-level intensity values in the image, where a lower GLN value correlates with a greater similarity in intensity values
GLCM	Inverse Variance	$inverse\ variance = \sum_{k=1}^{N_g-1} \frac{p_{x-y}(k)}{k^2}$	Note that k = 0 is skipped, as this would result in a division by 0.
NGTDM	Coarseness	$Coarseness = \frac{1}{\sum_{i=1}^{N_g} p_i s_i}$	Coarseness is a measure of average difference between the center voxel and its neighbourhood and is an indication of the spatial rate of change. A higher value indicates a lower spatial change rate and a locally more uniform texture.
GLSZM	Small Area Emphasis	$SAE = \frac{\sum_{i=1}^{N_g} \sum_{j=1}^{N_s} \frac{\mathbf{P}(i,j)}{j^2}}{N_z}$	a measure of the distribution of small size zones, with a greater value indicative of more smaller size zones and more fine textures.
GLDM	Small Dependence Emphasis	$SDE = \frac{\sum_{i=1}^{N_g} \sum_{j=1}^{N_d} \frac{\mathbf{P}(i,j)}{i^2}}{N_z}$	A measure of the distribution of small dependencies, with a

			greater value indicative of smaller dependence and less homogeneous textures.
GLCM	Idn	$$IDN = \sum_{k=0}^{N_g-1} \frac{p_{x-y}(k)}{1+\left(\frac{k}{N_g}\right)}$$	IDN (inverse difference normalized) is another measure of the local homogeneity of an image. Unlike Homogeneity1, IDN normalizes the difference between the neighboring intensity values by dividing over the total number of discrete intensity values.
GLDM	Gray Level Non Uniformity	$$GLN = \frac{\sum_{i=1}^{N_g}\left(\sum_{j=1}^{N_d}\mathbf{P}(i,j)\right)^2}{N_z}$$	Measures the similarity of gray-level intensity values in the image, where a lower GLN value correlates with a greater similarity in intensity values.
GLDM	Dependence Variance	$$DV = \sum_{i=1}^{N_g}\sum_{j=1}^{N_d} p(i,j)(j-\mu)^2,$$	Measures the variance in dependence size in the image.
GLDM	Dependence Non Uniformity Normalized	$$DNN = \frac{\sum_{j=1}^{N_d}\left(\sum_{i=1}^{N_g}\mathbf{P}(i,j)\right)^2}{N_z^2}$$	Measures the similarity of dependence throughout the image, with a lower value indicating more homogeneity among dependencies in the

			image. This is the normalized version of the DLN formula.
GLDM	Large Dependence Emphasis	$LDE = \frac{\sum_{i=1}^{N_g} \sum_{j=1}^{N_d} \mathbf{P}(i,j) j^2}{N_z}$	A measure of the distribution of large dependencies, with a greater value indicative of larger dependence and more homogeneous textures.
GLSZM	Large Area Emphasis	$LAE = \frac{\sum_{i=1}^{N_g} \sum_{j=1}^{N_s} \mathbf{P}(i,j) j^2}{N_z}$	LAE is a measure of the distribution of large area size zones, with a greater value indicative of more larger size zones and more coarse textures.
GLRLM	Run Entropy	$RE = -\sum_{i=1}^{N_g} \sum_{j=1}^{N_r} p(i,j\|\theta) \log_2(p(i,j\|\theta) + \epsilon)$	RE measures the uncertainty/randomness in the distribution of run lengths and gray levels. A higher value indicates more heterogeneity in the texture patterns.
GLRLM	Run Percentage	$RP = \frac{N_r(\theta)}{N_p}$	RP measures the coarseness of the texture by taking the ratio of number of runs and number of voxels in the ROI.
GLSZM	Large Area High Gray Level Emphasis	$LAHGLE = \frac{\sum_{i=1}^{N_g} \sum_{j=1}^{N_s} \mathbf{P}(i,j) i^2 j^2}{N_z}$	LAHGLE measures the proportion in the image of the

			joint distribution of larger size zones with higher gray-level values.
GLRLM	Run Length Non Uniformity Normalized	$RLNN = \frac{\sum_{j=1}^{N_r}\left(\sum_{i=1}^{N_g}\mathbf{P}(i,j\|\theta)\right)^2}{N_r(\theta)^2}$	RLNN measures the similarity of run lengths throughout the image, with a lower value indicating more homogeneity among run lengths in the image. This is the normalized version of the RLN formula.

Table S8. The performance parameters of five machine learning algorithms with MRI-based features and scores.

ML algorithms	ACC	SNS	SPC	PPV	NPV	AUC
LGR	0.777	0.751	0.807	0.836	0.753	0.867
SVM	0.772	0.783	0.758	0.795	0.775	0.866
RF	0.784	0.816	0.747	0.805	0.798	0.861
KNN	0.758	0.720	0.802	0.823	0.726	0.846
XGB	0.766	0.816	0.708	0.782	0.786	0.844

Supplementary Materials—Figures

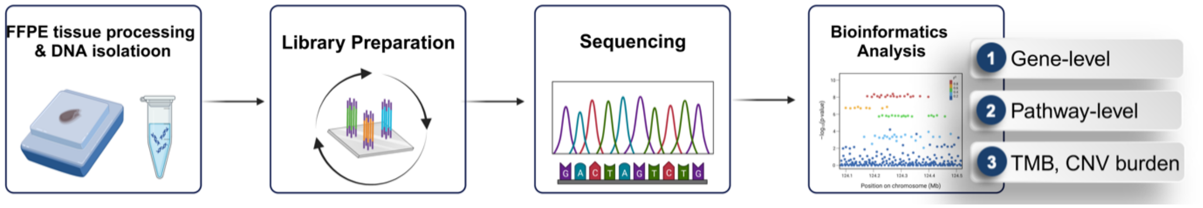

Figure S1. The pipeline of genomics data acquisition.

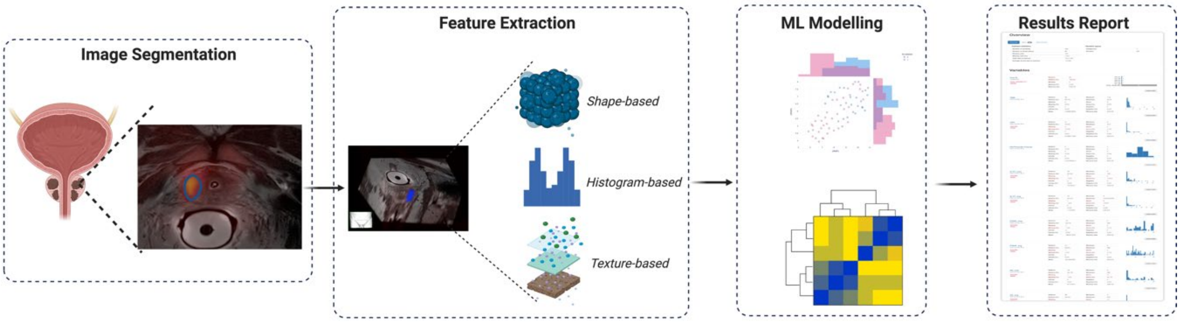

Figure S2. The workflow of radiomics and machine learning.

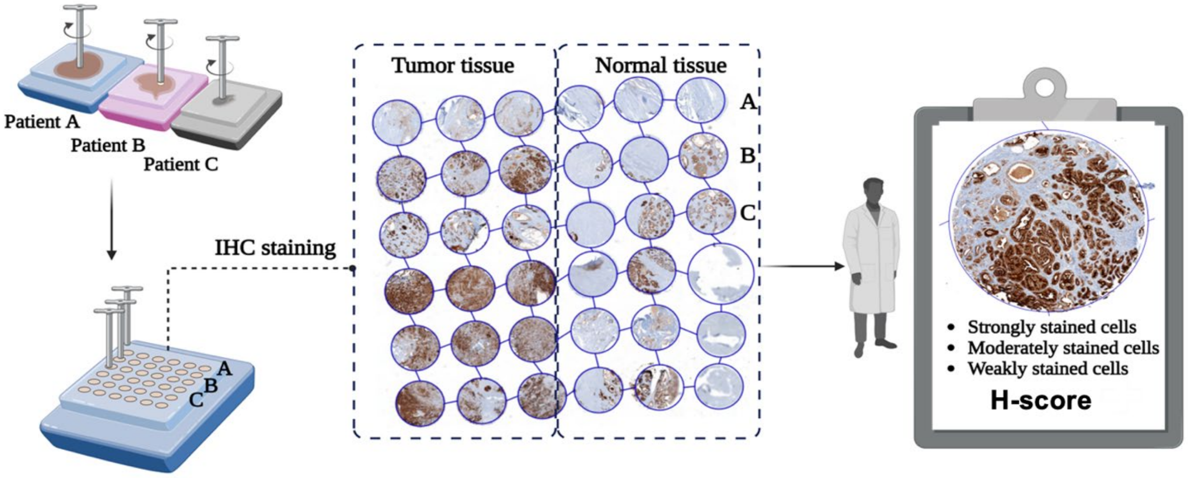

Figure S3. The working scheme for pathomics data acquisition.

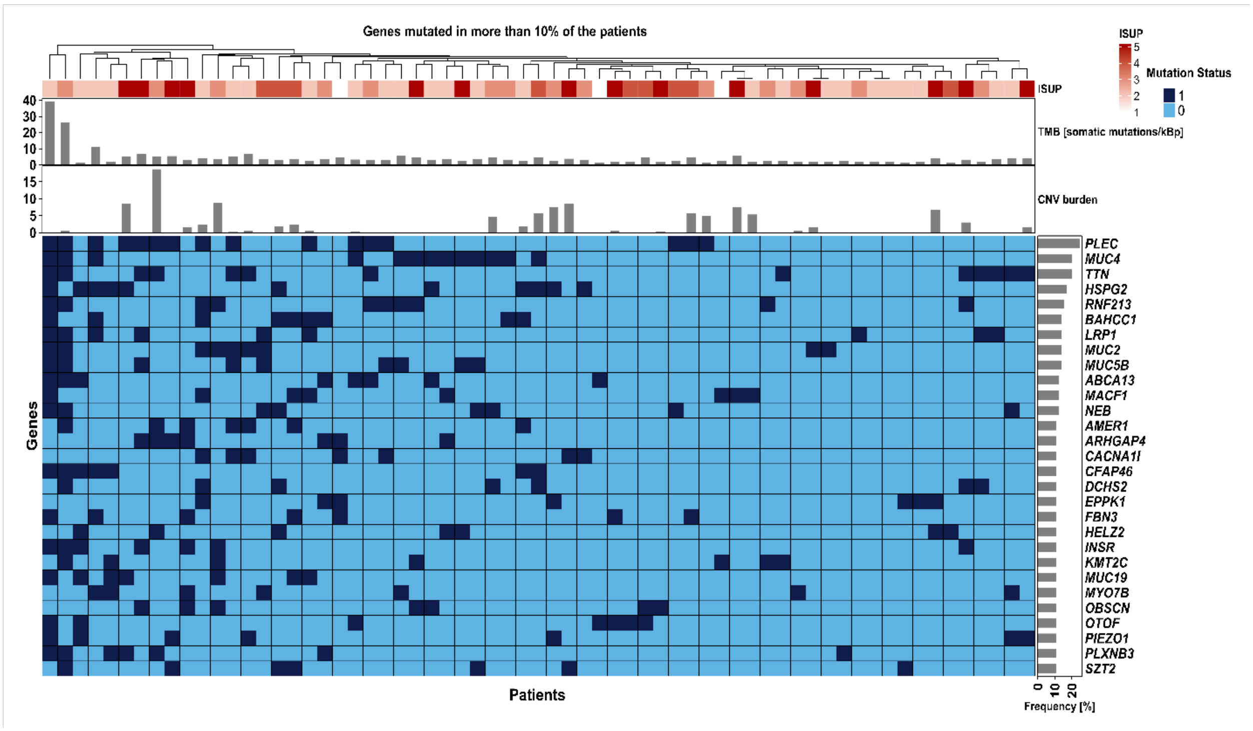

Figure S4: Mutation profile for genes with mutation frequency of ≥10% among the 65 PCa patients. Each row corresponds to a gene and each column represents one patient. The bar plot on the right side indicates the mutation frequency in descending order.

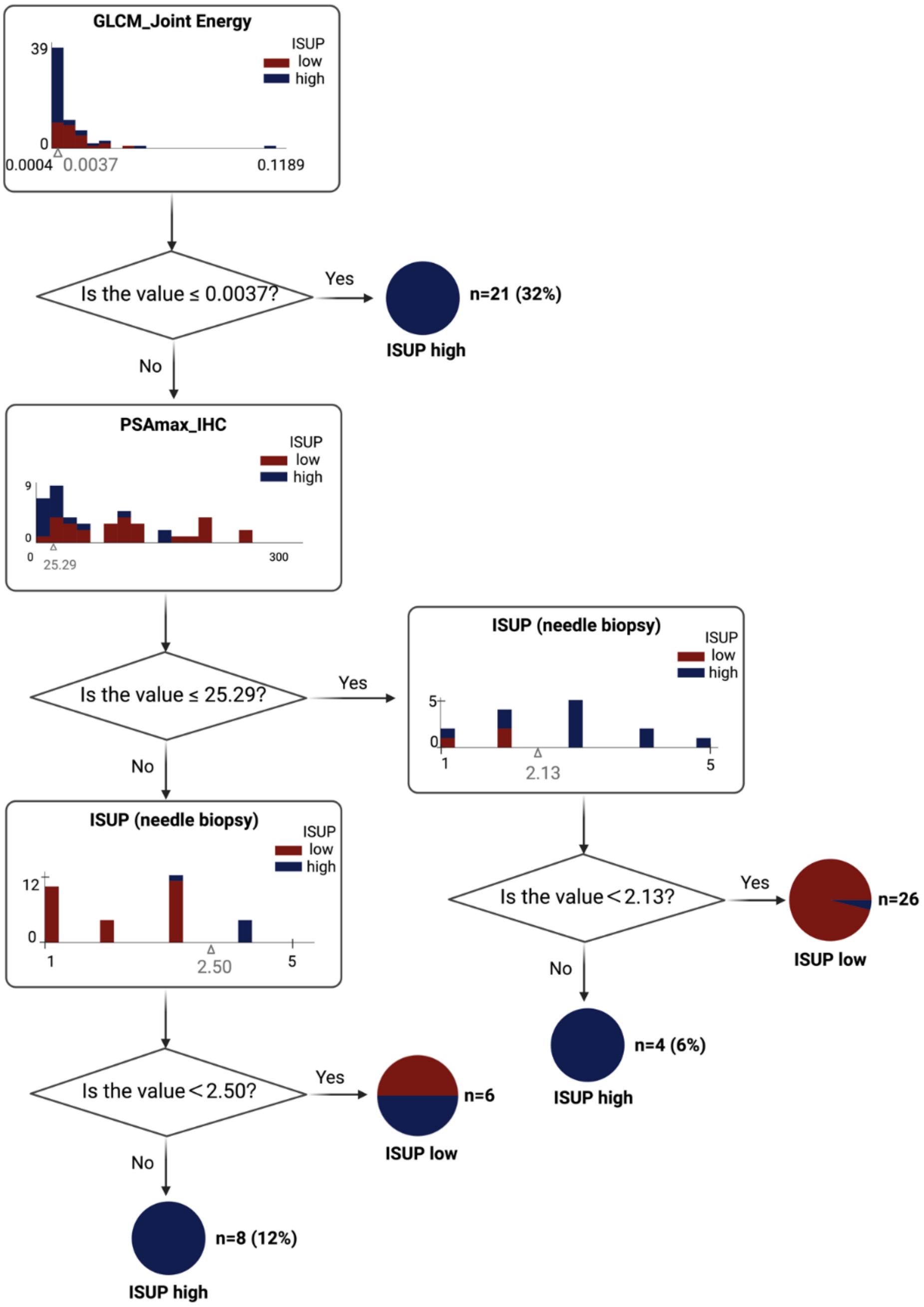

Figure S5: The machine learning (ML)-derived diagnostic workflow to select candidates for radical prostatectomy (RP) by the differentiation of high-risk PCa from low-risk PCa patients. Following the decision tree from the top, the urologist can discern the ISUP high from ISUP low PCa patients. In the rectangular boxes, the bar plot shows the distribution of each feature at the corresponding decision node during model training, where the y-axis represents the number of patients and the x-axis indicates the value of each feature.

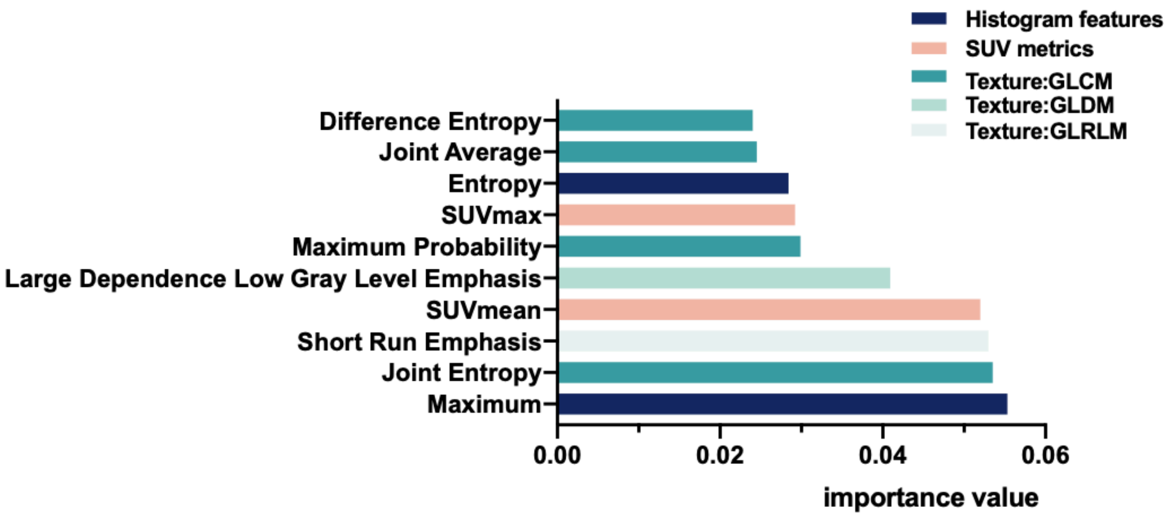

Figure S6. The 10 most important radiomics features that contribute most to the prediction of whole mount Gleason grading in the ML model based on permutation importance. The top ten important features are respectively Maximum, Joint Entropy, Short Run Emphasis, SUVmean, Large Dependence Low Gray Level Emphasis, Maximum Probability, SUVmax, Entropy, Joint Average, Difference Entropy in descending order of the permutation importance.

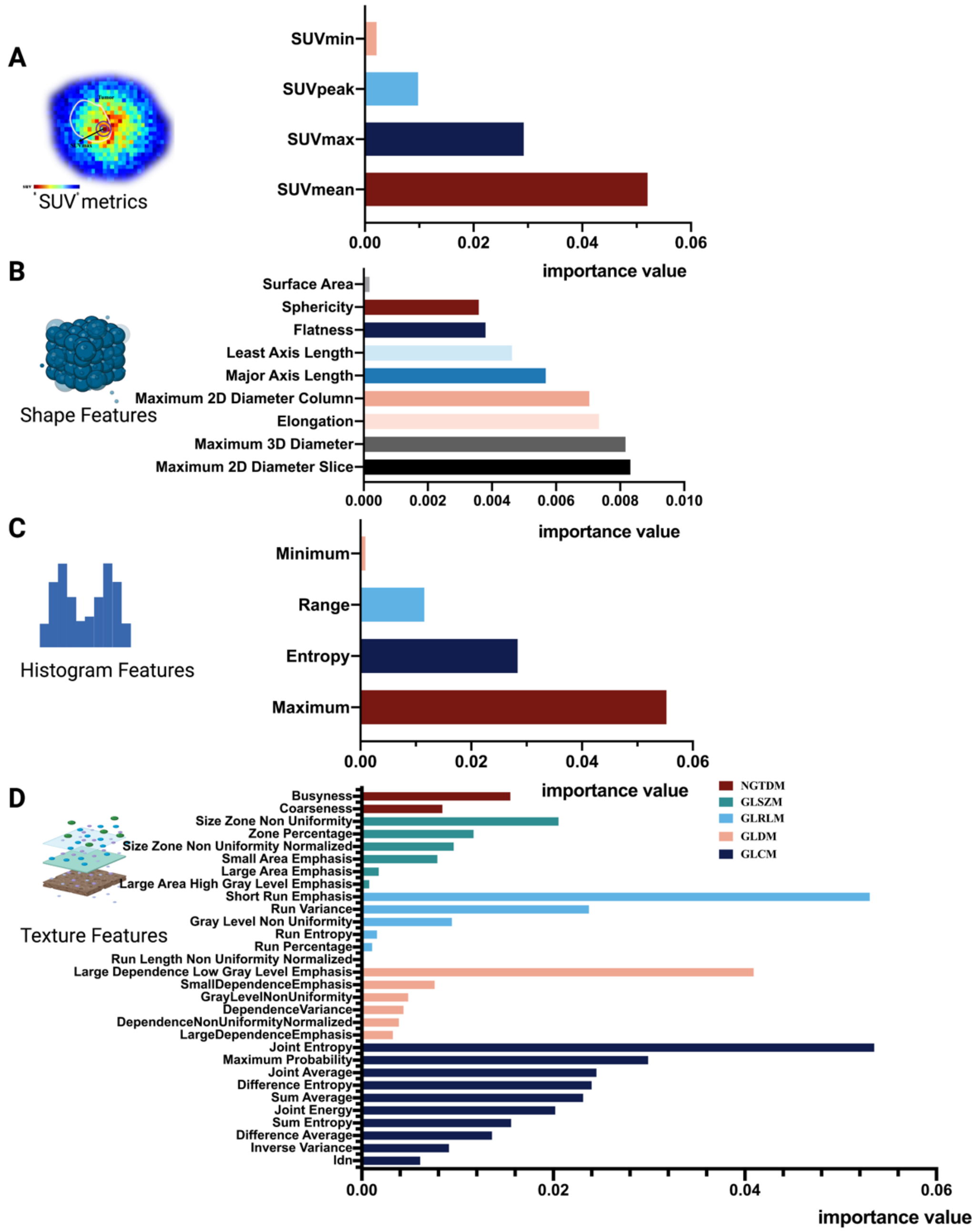

Figure S7. The subgroup analysis of key imaging features within each category according to permutation importance. A. Among SUV metrics, SUVmean, SUVmax, SUVpeak and SUVmin play a role in descending order in the ML model; B. Among shape features, Maximum 2D Maximum 2D diameter (Slice), Maximum 3D diameter, Elongation, Maximum 2D diameter (Column), Major Axis Length, Least Axis Length, Flatness, Sphericity, Surface Area are important features; C. Among histogram features, Maximum, Entropy, Range, Minimum contribute to the ML model; D. Among texture features, 2

NGTDM-based features, 6 GLSZM-based features, 6 GLRLM-based features, 6 GLDM-based features, 10 GLCM-based feature are of vital significance.

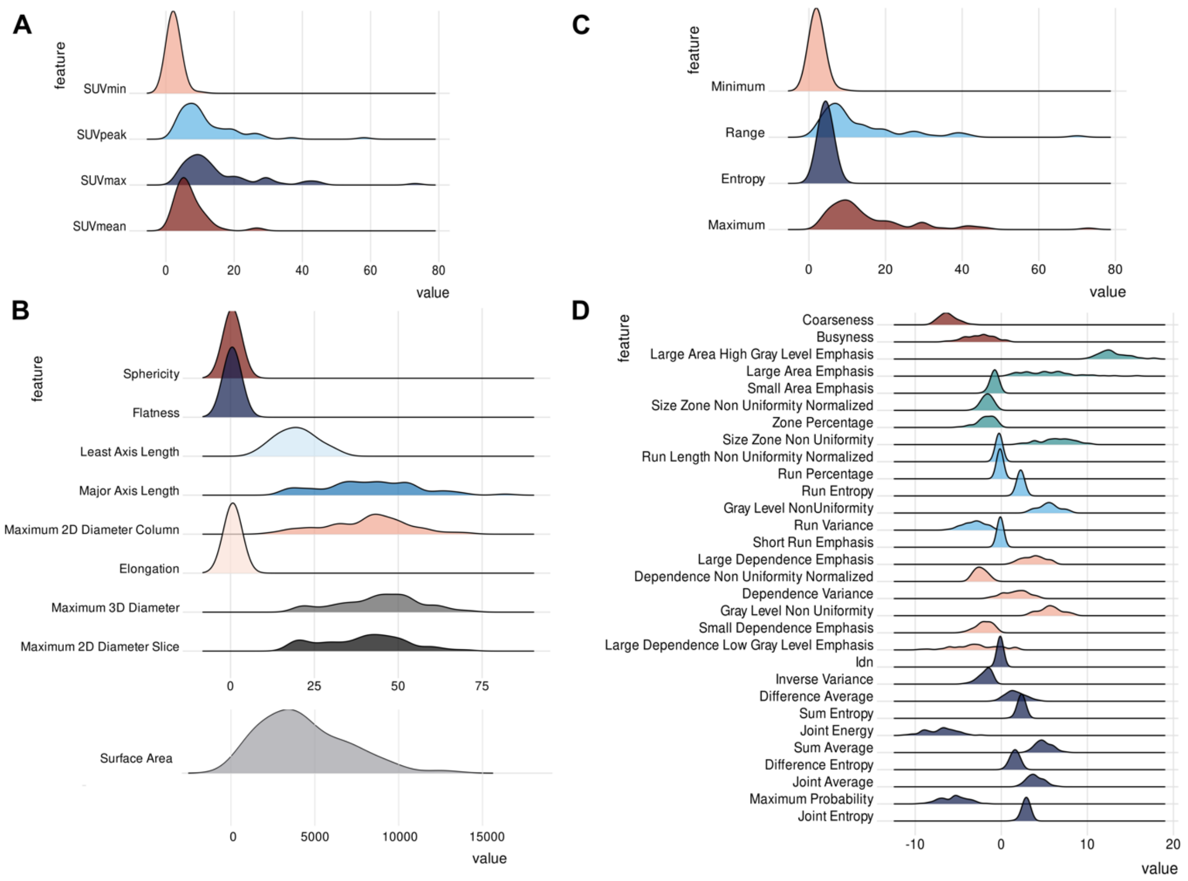

Figure S8. The distribution of important radiomic features based on permutation importance among 65 PCa patients. A. The distribution of 4 important SUV metrics. B. The distribution of 9 important shape features. Since the feature Surface Area has a significantly different range from the other shape features, it was separated. C. The distribution of 4 important histogram features. D. The distribution of 30 texture features in standardized values (log2).

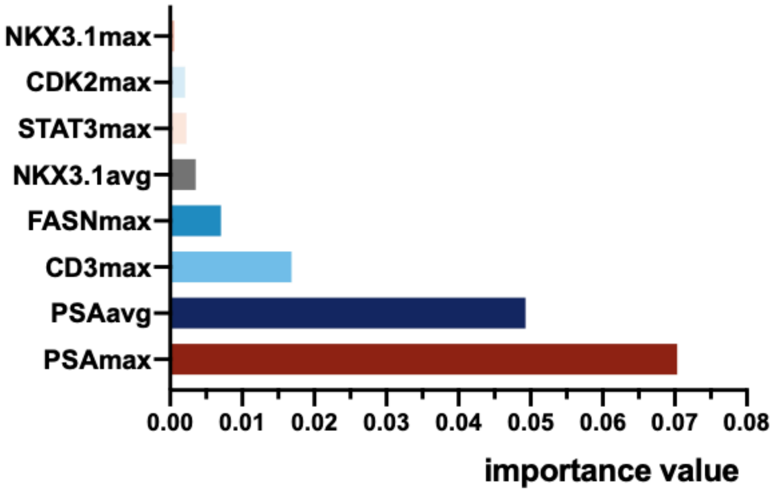

Figure S9. All the important pathomics features that contribute to the prediction of the whole mount Gleason grading. This bar plot showed these five features (PSA, CD3, FASN, NKX3.1, STAT3, CDK2) are the most contributing biomarkers to predict Gleason grading.

References

1. Li H, Durbin R: Fast and accurate short read alignment with Burrows–Wheeler transform. Bioinformatics 25:1754–1760, 2009

2. picard: A set of command line tools (in Java) for manipulating high-throughput sequencing (HTS) data and formats such as SAM/BAM/CRAM and VCF [Internet]. Github[cited 2024 Mar 4] Available from: https://github.com/broadinstitute/picard

3. Fang LT, Afshar PT, Chhibber A, et al: An ensemble approach to accurately detect somatic mutations using SomaticSeq. Genome Biol 16:197, 2015

4. Kim S, Scheffler K, Halpern AL, et al: Strelka2: fast and accurate calling of germline and somatic variants. Nat Methods 15:591–594, 2018

5. Lai Z, Markovets A, Ahdesmaki M, et al: VarDict: a novel and versatile variant caller for next-generation sequencing in cancer research. Nucleic Acids Res 44:e108, 2016

6. Cibulskis K, Lawrence MS, Carter SL, et al: Sensitive detection of somatic point mutations in impure and heterogeneous cancer samples. Nat Biotechnol 31:213–219, 2013

7. Larson DE, Harris CC, Chen K, et al: SomaticSniper: identification of somatic point mutations in whole genome sequencing data. Bioinformatics 28:311–317, 2012

8. Wilm A, Aw PPK, Bertrand D, et al: LoFreq: a sequence-quality aware, ultra-sensitive variant caller for uncovering cell-population heterogeneity from high-throughput sequencing datasets. Nucleic Acids Res 40:11189–11201, 2012

9. Fan Y, Xi L, Hughes DST, et al: MuSE: accounting for tumor heterogeneity using a sample-specific error model improves sensitivity and specificity in mutation calling from sequencing data. Genome Biol 17:178, 2016

10. Koboldt DC, Zhang Q, Larson DE, et al: VarScan 2: somatic mutation and copy number alteration discovery in cancer by exome sequencing. Genome Res 22:568–576, 2012

11. McLaren W, Gil L, Hunt SE, et al: The Ensembl Variant Effect Predictor. Genome Biol 17:122, 2016

12. Rogers MF, Shihab HA, Mort M: EVE: An ensemble of CADD-like scores for enhancing variant effect prediction. Bioinformatics 34:4077–4084, 2018

13. Rentzsch P, Witten D, Cooper GM, et al: CADD: predicting the deleteriousness of variants throughout the human genome. Nucleic Acids Res 47:D886–D894, 2019

14. Adzhubei IA, Schmidt S, Peshkin L, et al: A method and server for predicting damaging missense mutations. Nat Methods 7:248–249, 2010

15. Kanehisa M, Goto S: KEGG: kyoto encyclopedia of genes and genomes. Nucleic Acids Res 28:27–30, 2000

16. Talevich E, Shain AH, Botton T, et al: CNVkit: Genome-Wide Copy Number Detection and Visualization from Targeted DNA Sequencing. PLoS Comput Biol 12:e1004873, 2016

17. Evans JC, Malhotra M, Cryan JF, et al: The therapeutic and diagnostic potential of the prostate specific membrane antigen/glutamate carboxypeptidase II (PSMA/GCPII) in cancer and neurological disease. Br J Pharmacol 173:3041–3079, 2016

18. McBean GJ, Aslan M, Griffiths HR, et al: Thiol redox homeostasis in neurodegenerative disease. Redox Biol 5:186–194, 2015

19. Caromile LA, Dortche K, Rahman MM, et al: PSMA redirects cell survival signaling from the MAPK to the PI3K-AKT pathways to promote the progression of prostate cancer [Internet]. Sci Signal 10, 2017Available from: http://dx.doi.org/10.1126/scisignal.aag3326

20. He Y, Xu W, Xiao Y-T, et al: Targeting signaling pathways in prostate cancer: mechanisms and clinical trials. Signal Transduction and Targeted Therapy 7:1–31, 2022

21. Silver DA, Pellicer I, Fair WR, et al: Prostate-specific membrane antigen expression in normal and malignant human tissues. Clin Cancer Res 3:81–85, 1997

22. Zalcman N, Canello T, Ovadia H, et al: Androgen receptor: a potential therapeutic target for glioblastoma. Oncotarget 9:19980–19993, 2018

23. Bianchi VE, Rizzi L, Bresciani E, et al: Androgen Therapy in Neurodegenerative Diseases. J Endocr Soc 4:bvaa120, 2020

24. Pike CJ, Nguyen T-VV, Ramsden M, et al: Androgen cell signaling pathways involved in neuroprotective actions. Horm Behav 53:693–705, 2008

25. Ramalingam S, Ramamurthy VP, Njar VCO: Dissecting major signaling pathways in prostate cancer development and progression: Mechanisms and novel therapeutic targets. J Steroid Biochem Mol Biol 166:16–27, 2017

26. Shtivelman E, Beer TM, Evans CP: Molecular pathways and targets in prostate cancer. Oncotarget 5:7217–7259, 2014

27. Morgan C, Jenkins SA, Kynaston HG, et al: The role of adhesion molecules as biomarkers for the aggressive prostate cancer phenotype. PLoS One 8:e81666, 2013

28. Guccini I, Revandkar A, D'Ambrosio M, et al: Senescence Reprogramming by TIMP1 Deficiency Promotes Prostate Cancer Metastasis. Cancer Cell 39:68-82.e9, 2021

29. Pisano C, Tucci M, Di Stefano RF, et al: Interactions between androgen receptor signaling and other molecular pathways in prostate cancer progression: Current and future clinical implications. Crit Rev Oncol Hematol 157:103185, 2021

30. Shah A, Shah AA, K N, et al: Mechanistic targets for BPH and prostate cancer-a review. Rev Environ Health 36:261–270, 2021

31. Tran MGB, Bibby BAS, Yang L, et al: Independence of HIF1a and androgen signaling pathways in prostate cancer. BMC Cancer 20:469, 2020

32. Hartley A, Ahmad I: The role of PPARγ in prostate cancer development and progression. Br J Cancer 128:940–945, 2022

33. Zielinski RR, Eigl BJ, Chi KN: Targeting the apoptosis pathway in prostate cancer. Cancer J 19:79–89, 2013

34. Awwad HM, Geisel J, Obeid R: The role of choline in prostate cancer. Clin Biochem 45:1548–1553, 2012

35. Poulose N, Amoroso F, Steele RE, et al: Genetics of lipid metabolism in prostate cancer. Nat Genet 50:169–171, 2018

36. Sena LA, Denmeade SR: Fatty Acid Synthesis in Prostate Cancer: Vulnerability or Epiphenomenon? Cancer Res 81:4385–4393, 2021

37. Ahmad F, Cherukuri MK, Choyke PL: Metabolic reprogramming in prostate cancer. Br J Cancer 125:1185–1196, 2021

38. Frégeau-Proulx L, Lacouture A, Berthiaume L, et al: Multiple metabolic pathways fuel the truncated tricarboxylic acid cycle of the prostate to sustain constant citrate production and secretion. Mol Metab 62:101516, 2022

39. Heidegger I, Kern J, Ofer P, et al: Oncogenic functions of IGF1R and INSR in prostate cancer include enhanced tumor growth, cell migration and angiogenesis. Oncotarget 5:2723–2735, 2014

40. Luthold C, Hallal T, Labbé DP, et al: The Extracellular Matrix Stiffening: A Trigger of Prostate Cancer Progression and Castration Resistance? [Internet]. Cancers 14, 2022Available from: http://dx.doi.org/10.3390/cancers14122887

41. Figel S, Gelman IH: Focal adhesion kinase controls prostate cancer progression via intrinsic kinase and scaffolding functions. Anticancer Agents Med Chem 11:607–616, 2011

42. Zhang H, Kong Q, Wang J, et al: Complex roles of cAMP-PKA-CREB signaling in cancer. Exp Hematol Oncol 9:32, 2020

43. Bailey CL, Kelly P, Casey PJ: Activation of Rap1 promotes prostate cancer metastasis. Cancer Res 69:4962–4968, 2009

44. Aksoy O, Pencik J, Hartenbach M, et al: Thyroid and androgen receptor signaling are antagonized by μ-Crystallin in prostate cancer. Int J Cancer 148:731–747, 2021

45. Xu Y, Song G, Xie S, et al: The roles of PD-1/PD-L1 in the prognosis and immunotherapy of prostate cancer. Mol Ther 29:1958–1969, 2021

46. Shahzad MH, Feng L, Su X, et al: Neutrophil Extracellular Traps in Cancer Therapy Resistance [Internet]. Cancers 14, 2022Available from: http://dx.doi.org/10.3390/cancers14051359

47. Silberstein J, Downs T, Lakin C, et al: HIV and prostate cancer: a systematic review of the literature. Prostate Cancer Prostatic Dis 12:6–12, 2009

48. Zhao S, Zhang Y, Zhang Q, et al: Toll-like receptors and prostate cancer. Front Immunol 5:352, 2014

49. Lai H, Zeng D, Liu C, et al: Selenium-containing ruthenium complex synergizes with natural killer cells to enhance immunotherapy against prostate cancer via activating TRAIL/FasL signaling. Biomaterials 219:119377, 2019
